# Effective Blocking of the *White* Enhancer Requires Cooperation between Two Main Mechanisms Suggested for the Insulator Function

**DOI:** 10.1371/journal.pgen.1003606

**Published:** 2013-07-04

**Authors:** Olga Kyrchanova, Oksana Maksimenko, Viacheslav Stakhov, Tatyana Ivlieva, Alexander Parshikov, Vasily M. Studitsky, Pavel Georgiev

**Affiliations:** 1 Group of Transcriptional Regulation, Institute of Gene Biology, Russian Academy of Sciences, Moscow, Russia; 2 Department of the Control of Genetic Processes, Institute of Gene Biology, Russian Academy of Sciences, Moscow, Russia; 3 Laboratory of Epigenetic Regulation of Transcription, Institute of Gene Biology, Russian Academy of Sciences, Moscow, Russia; 4 Department of Pharmacology, UMDNJ–Robert Wood Johnson Medical School, Piscataway, New Jersey, United States of America; University of Cambridge, United Kingdom

## Abstract

Chromatin insulators block the action of transcriptional enhancers when interposed between an enhancer and a promoter. In this study, we examined the role of chromatin loops formed by two unrelated insulators, *gypsy* and Fab-7, in their enhancer-blocking activity. To test for this activity, we selected the *white* reporter gene that is activated by the eye-specific enhancer. The results showed that one copy of the *gypsy* or Fab-7 insulator failed to block the eye enhancer in most of genomic sites, whereas a chromatin loop formed by two *gypsy* insulators flanking either the eye enhancer or the reporter completely blocked *white* stimulation by the enhancer. However, strong enhancer blocking was achieved due not only to chromatin loop formation but also to the direct interaction of the *gypsy* insulator with the eye enhancer, which was confirmed by the 3C assay. In particular, it was observed that Mod(mdg4)-67.2, a component of the *gypsy* insulator, interacted with the Zeste protein, which is critical for the eye enhancer–*white* promoter communication. These results suggest that efficient enhancer blocking depends on the combination of two factors: chromatin loop formation by paired insulators, which generates physical constraints for enhancer–promoter communication, and the direct interaction of proteins recruited to an insulator and to the enhancer–promoter pair.

## Author Summary

The mechanism underlying enhancer blocking by insulators is unclear. Current models suggest that insulator proteins block enhancers either by formation of chromatin loops or by direct interaction with protein complexes bound to the enhancers and promoters. Here, we tested the role of a chromatin loop in blocking the activity of two Drosophila insulators, *gypsy* and Fab-7. Both insulators failed to effectively block the interaction between the eye enhancer and the *white* promoter at most of genomic sites. Insertion of an additional *gypsy* copy either upstream of the eye enhancer or downstream from the *white* gene led to complete blocking of the enhancer–promoter communication. In contrast, flanking of the eye enhancer by Fab-7 insulators only weakly improved enhancer blocking. Such a difference in enhancer blocking may be explained by finding that Mod(mdg4)-67.2, a component of *gypsy* insulator, directly interacts with the Zeste protein, which is critical for enhancer–promoter communication in the *white* gene.

## Introduction

The complexity of regulatory systems in higher eukaryotes, featuring many distantly located enhancers that nonetheless properly activate the target, has promoted the hypothesis that the action of enhancers should be restricted by elements called insulators. Initially, insulators were regarded as genomic regulatory elements (nucleoprotein complexes) that have two characteristic properties: they can block the action of an enhancer on a promoter when interposed between them and can protect the transgenes they flank from chromosomal position effects (for reviews, see [1]–[7]). However, recent results of studies on insulators in transgenic *Drosophila* lines [8]–[12], genome-wide identification of biding sites for insulator proteins by ChIP-on-ChIP and ChIP-seq [13]–[17], analysis of locus architecture by different variants of chromosome conformation capture technology [18]–[19], and genome-wide analysis of interaction between CTCF sites by paired-end tag (PET) approach, ChIA-PET [20], and Hi-C technique [21], [22] suggest that insulators are mainly involved in organization of long-distance specific interactions between remote genome regions such as enhancers and promoters, different promoters, or multiple regulatory elements.

Well-characterized insulators in *Drosophila* include the scs and scs' sequences from the 87A heat shock locus [23], [24]; the Mcp, Fab-7 and Fab-8 insulators from the *Abd-B* regulatory region [25]–[29]; the SF1 insulator from the Antennapedia complex [30]; the IdefixU3 insulator [31]; the Wari insulator located at the 3′ side of the *white* gene [10]; and the insulator sequences associated with the Su(Hw) protein [32]–[36]. Today, there are two basic models explaining how insulators block the activity of enhancers [1], [2], [4], [6], [37]. The decoy model suggests that the insulator complex binds to an enhancer or a promoter complex to neutralize it or traps its vital component(s). The alternative model suggests that the interaction between insulators results in the formation of chromatin loops that constrain interaction between an enhancer and a promoter located on the opposite sides of the insulator. The latter model is indirectly supported by the ability of the insulators to specifically interact over large distances [11], [12], [20]–[22], [38]–[40]. However, there are only a few pieces of indirect experimental evidence supporting the model that a loop formed by interacting insulators is essential for enhancer blocking [41]–[46].

Bondarenko et al. (2003) used a bacterial enhancer–promoter pair and a pair of *lac* operators (*lac*O) that mimicked eukaryotic insulators [42], [43]. It was shown in an *in vitro* transcription assay that the enhancer action was blocked when the interacting *lac*O copies formed two closed loops, one with the enhancer and the other with the promoter. This finding suggests that if DNA looping alone is sufficient to suppress the enhancer activity in an *in vitro* model system, it may as well play an important role in eukaryotic cells. Ameres et al. (2005) examined the expression of a reporter gene in HeLa cells transfected with the plasmid in which the SV40 enhancer was placed downstream of the reporter gene [41]. The SV40 enhancer was flanked by two boxes, each consisting of seven repeats of the tetR element. When the chimeric protein consisting of the tetR protein and a dimerization domain bound to tetR elements, the dimerized proteins formed a 344-bp chromatin loop containing the SV40 enhancer. As a result, this enhancer was blocked, with consequent reduction of the reporter gene expression, which suggested a role for the loop in preventing the interaction between the SV40 enhancer and the promoter. However, alternative mechanisms of SV40 enhancer blocking cannot be excluded. For example, the small chromatin loop may interfere with proper binding of transcription factors to the enhancer. In the study by Hou et al. (2008), a CTCF-dependent insulator ectopically inserted between the beta-globin locus control region (LCR) and downstream genes was found to function as an enhancer blocker and form an aberrant loop with the endogenous CTCF region located upstream of the LCR [46]. However, these authors did not perform experiments to show that the ectopic insulator could not block LCR in the absence of the loop formation with the endogenous insulator and, therefore, failed to obtain direct evidence for the role of chromatin loop formation in enhancer blocking.

For this reason, we established a *Drosophila* transgenic model system in order to test whether isolation of either an enhancer or the reporter *white* gene in a loop formed by *gypsy* or Fab-7 insulators can block the enhancer–promoter communication.

The *gypsy* insulator, the strongest and the best studied in *Drosophila*, contains 12 consecutive degenerate direct repeats of the binding motif for the zinc-finger protein Su(Hw), which is indispensable for the insulator function [32], [33]. The Su(Hw) protein also associates with hundreds of non-*gypsy* regions that do not contain clustered Su(Hw)-binding sites, with the vast majority of them carrying a single copy of the corresponding sequence [13], [14], [36]. Su(Hw) interacts with three other components of the *gypsy* insulator, Mod(mdg4)-67.2 [47], [48], CP190 [49], and E(y)2 [50]. Based on the results of genetic interactions, it has been suggested that Mod(mdg4)-67.2 and CP190 are essential for the enhancer-blocking activity of Su(Hw) insulators. Mod(mdg4)-67.2 interacts with Su(Hw) through its carboxy-terminal domain [47], [48], [51]. The BTB domain is located at the N-terminus of Mod(mdg4)-67.2 and mediates homo-multimerization [52]. There are many evidences of functional distant interactions between the *gypsy* insulators [9], [11], [53], which were recently confirmed by the 3C method [54].

Among insulators found in the regulatory region of *Abd-B*, the best characterized is Fab-7 located between the iab-6 and iab-7 *cis*-regulatory domains [55]. Mutations that inactivate Fab-7 lead to the fusion of the iab-6 and iab-7 domains, and this disrupts the specification of PS11 [56]–[58]. Previously we found that interaction between paired two copies of the Fab-7 insulator can support long-distance enhancer-promoter interactions [59]. It was also found that Fab-7 insulators, similar to *gypsy* insulators [11], can support interactions across several megabases [12].

In previous studies [9], [28], [33], [45], [59], [60]–[62], the activities of *gypsy* and Fab-7 insulators were mainly tested in transgenic lines with the *yellow* and *white* genes as reporters that allowed changes in gene expression to be assayed by simple phenotypic analysis. It was found that one copy of the *gypsy* insulator completely blocked the communication between the *yellow* enhancers and the promoter [33], [60], [62]. Genetic studies on transgenic lines carrying different mutations in the *yellow* promoter suggested that the *gypsy* insulator directly interacted with the *yellow* promoter [63]. Placing the *yellow* enhancers in a 10-kb chromatin loop formed by the *gypsy* insulators led to neutralization of enhancer blocking [62], providing evidence against the role of a chromatin loop formed by the insulators in enhancer blocking.

Therefore, we used the *white* model system to test the role of the chromatin loop in the enhancer-blocking activity of the *gypsy* and *Fab-7* insulators. Recently we found that the *white* gene contains the insulator, named Wari, located downstream of the polyadenylation signal [10]. This insulator can interact equally well with another copy of Wari and with unrelated Su(Hw) insulators [9], [10]. It was shown that the interaction between the Wari and *gypsy* insulators strongly improves enhancer blocking. To test whether a single copy of *gypsy* or Fab-7 insulator can block the eye enhancer, we deleted the Wari insulator from the *white* gene.

We found that one copy of the *gypsy* or Fab-7 insulator failed to affect the eye enhancer activity in most of the transgenic lines tested. At the same time, the insertion of two *gypsy* insulators on both sides of the eye enhancer or the *white* gene completely blocked the enhancer–promoter communication in all transgenic lines. In contrast, flanking the eye enhancer by Fab-7 insulators only slightly contributed to the blocking of enhancer–promoter communication. Such a difference in the ability to block the eye enhancer between the pairs of *gypsy* and Fab-7 insulators is explained by the finding that the *gypsy* insulator can directly interact with the enhancer. In particular, Mod(mdg4)-67.2 interacts with Zeste and can interfere with its activity in supporting enhancer–promoter communication at the *white* gene.

## Results

### The *gypsy* insulator fails to block the eye enhancer in more than 70% of transgenic lines

**Figure 1:**
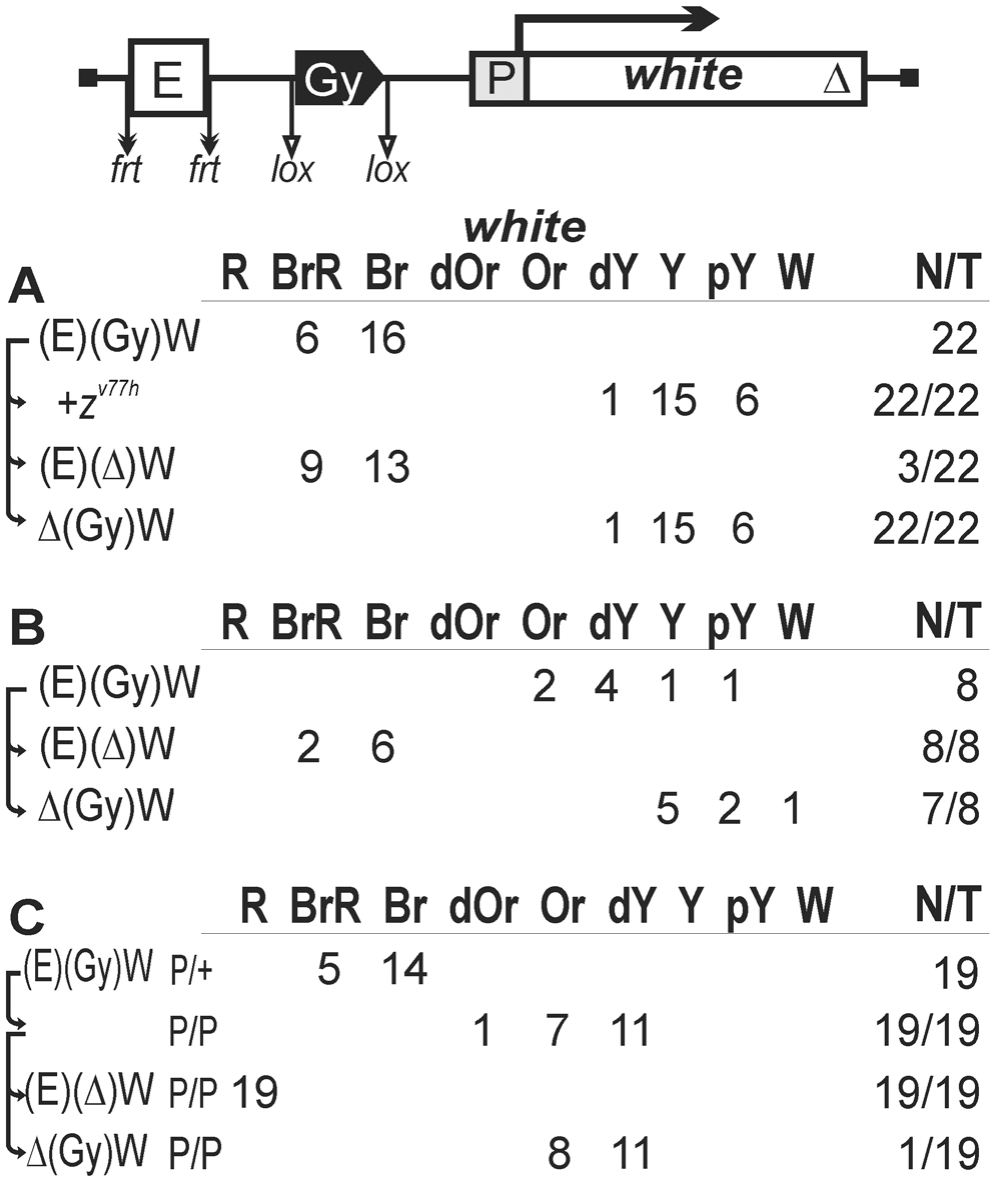
Testing the enhancer-blocking activity of the *gypsy* insulator in one copy. Transgenic lines were grouped into those in which the *gypsy* insulator displayed (A) weak or (B) strong enhancer-blocking activity. (C) Homozygous transgenic lines in which the *gypsy* insulator displayed a weak enhancer-blocking activity. In the reductive scheme of the transgenic construct used in the assay, the *white* gene is shown as white box with an arrow indicating the direction of transcription; the triangle indicates deletion of the Wari insulator located at the 3′ end of the *white* gene; downward arrows indicate target sites for Flp recombinase (*frt*) or Cre recombinase (*lox*); the same sites in construct names are denoted by parentheses; the eye enhancer (E) is shown as white rectangle; the direction of the *gypsy* insulator (Gy) is indicated by the apex of the pentagon. The numbers of transgenic lines with different levels of *white* pigmentation in the eyes are indicated. Arrows indicate the excision of an element to produce the derivative transgenic lines. Wild-type *white* expression determined the bright red eye color (R); in the absence of *white* expression, the eyes were white (W). Intermediate levels of pigmentation, with the eye color ranging from pale yellow (pY), through yellow (Y), dark yellow (dY), orange (Or), dark orange (dOr), and brown (Br) to brownish red (BrR), reflect the increasing levels of *white* expression. N is the number of lines in which flies acquired a new eye color phenotype by deletion (Δ) of the specified DNA fragment; T is the total number of lines examined for each particular construct. *z^v77h^*, a null-mutation of the *zeste* gene.

To establish a model system for testing the role of a chromatin loop in insulation, we used the *white* reporter gene that is stimulated by a tissue-specific enhancer in the eyes. The level of eye pigmentation is a sensitive indicator of the amount of *white* transcription. To test the enhancer-blocking activity of one copy of the *gypsy* insulator in different genomic positions, we deleted the Wari insulator from the *white* gene (W^Δ^). The *gypsy* insulator flanked by lox sites was inserted between the eye enhancer flanked by frt sites and the *white* gene (Figure 1A). Parentheses in construct designations and short downward arrows in the schemes indicate the elements flanked by lox or frt sites for in vivo excision by crossing, as outlined in Materials and Methods. Such excisions are denoted by “Δ” in the primary (expression) data. Comparing eye phenotypes in the transgenic lines before and after deletion of either the eye enhancer or the *gypsy* insulator allowed estimation of their contribution to *white* expression. Since it was shown [64] that the eye enhancer can initiate transcription in the direct orientation, we inserted the eye enhancer in either direct (Ee) or reverse orientation (Ee^R^). In Figure 1, we combined the results obtained with transgenic lines carrying both constructs, because they displayed a similar range of phenotypes.

**Figure 2:**
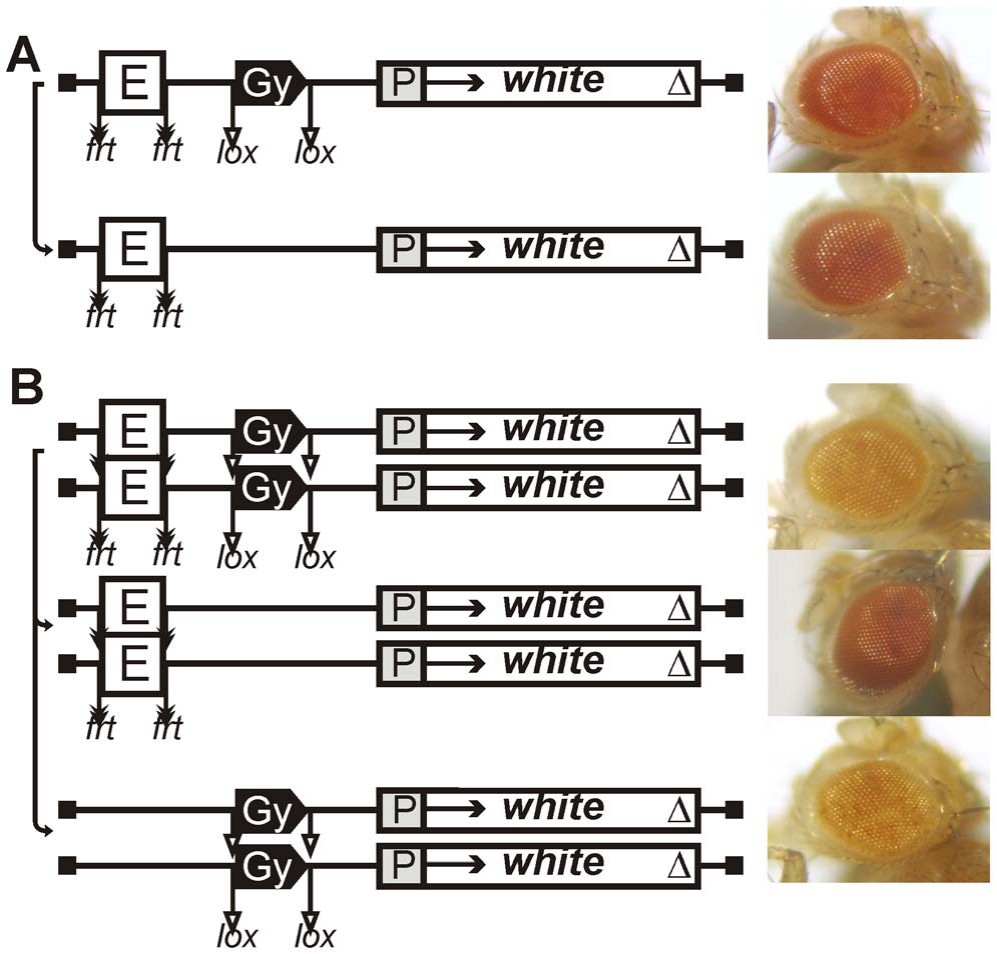
Eye phenotypes in flies from the transgenic line with the *gypsy* insulator in one copy. (A) heterozygous (P/+) or (B) homozygous (P/P) for the construct and in flies after deletion of either the eye enhancer or the *gypsy* insulator. Other designations are as in Figure 1.

In 22 out of 34 transgenic lines, males heterozygous for the construct had high levels of eye pigmentation (from brown to brown-red) that decreased significantly after deletion of the eye enhancer (Figures 1A, 2A). These results suggest that the eye enhancer can stimulate *white* expression across the *gypsy* insulator. Moreover, deletion of the *gypsy* insulator slightly changed eye pigmentation in only 3 out of 22 tested lines, indicating that *gypsy* failed to block the eye enhancer. To test for the functional role of Zeste in *white* stimulation by the eye enhancer, we crossed the transgenic lines into the background of *z^v77h^*, a null mutation of the *zeste* gene [65]. As a result, we observed that *z^v77h^* strongly reduced eye pigmentation to the same level as did the deletion of the eye enhancer (Figure 1A), indicating that Zeste is critical for the eye enhancer activity. After deletion of the *gypsy* insulator, *z^v77h^* still reduced *white* expression in transgenic lines (Figure S1A). However, *z^v77h^* did not influence *white* phenotypes in transgenic lines carrying derivative constructs with the deleted eye enhancer. These results suggest that Zeste is essential for the eye enhancer activity even when it is located close to the *white* promoter.

Four transgenic lines had yellow eyes that did not change in color after deletion of the *gypsy* insulator, indicating that the eye enhancer was inactive in these lines (data not shown). In the remaining eight lines (Figure 1B), the deletion of *gypsy* led to change in eye color from yellow to brown, suggesting that this insulator could effectively block the enhancer–promoter communication. Thus, the *gypsy* insulator proved to block the eye enhancer in only 8 out of 30 transgenic lines (27%).

Next, we examined eye color in flies of 19 homozygous transgenic lines in which the *gypsy* insulator failed to block the eye enhancer (Figure 1C). Unexpectedly, we found that flies carrying the homozygous transgene had lighter eyes than flies with the heterozygous transgene (Figures 1C, 2B). The deletion of the eye enhancer did not change eye pigmentation in lines homozygous for the transgene, indicating that the *gypsy* insulator completely blocked the eye enhancer in these lines.

Taken together, these results suggest that one copy of the *gypsy* insulator failed to block the eye enhancer–*white* promoter communication in more than 70% of the transgenic lines. However, pairing between the *gypsy* insulators located on homologous chromosomes restricted the eye enhancer activity.

### Fab-7 has a weak enhancer-blocking activity in most genomic sites of transgene insertion

As a second model insulator, we selected the well-described Fab-7 insulator that supports specific long-distance interactions [12]. As shown in previous experiments with transgenic lines carrying constructs with the 1.2-kb Fab-7 insulator inserted between the eye enhancer and the *white* promoter, flies in approximately half of these lines had relatively light eyes indicating effective blocking of the eye enhancer by the Fab-7 insulator [25], [61]. However, it was not proved that the eye enhancer was functional in these lines, since the Wari insulator located at the 3′ end of the *white* gene could also improve the activity of Fab-7.

**Figure 3:**
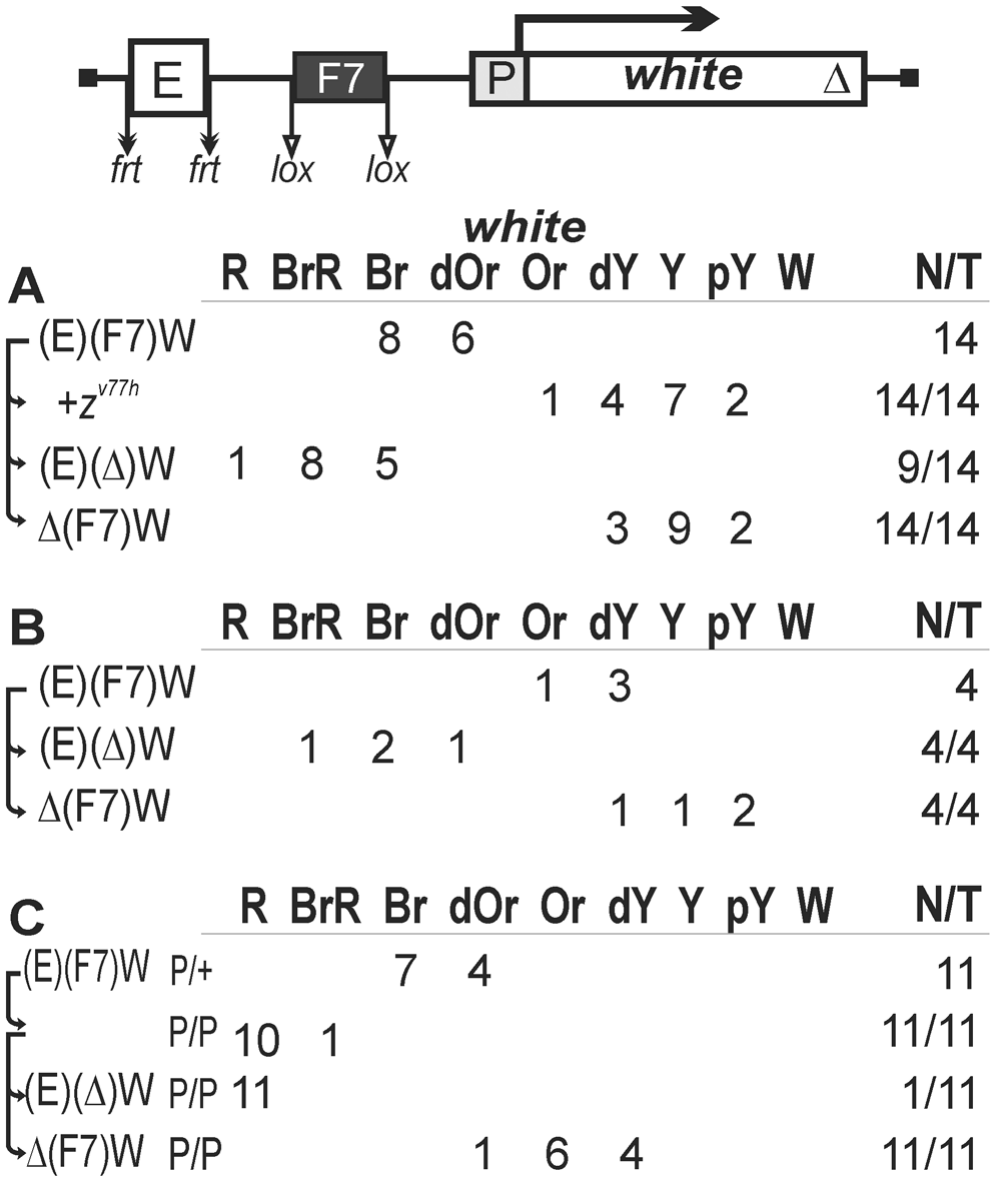
The enhancer-blocking activity of the Fab-7 insulator in one copy. Transgenic lines were grouped into those in which the Fab-7 insulator (F7, black rectangle) displayed (A) weak or (B) strong enhancer-blocking activity. (C) Homozygous transgenic lines in which the Fab-7 insulator displayed a weak enhancer-blocking activity. Other designations are as in Figure 1.

For these reasons, we again tested the enhancer-blocking ability of one Fab-7 copy in the construct where it was flanked by lox sites and inserted between the eye enhancer (flanked by frt sites) and the *white* gene (Figure 3). In 14 transgenic lines, flies had extensive eye pigmentation ranging from dark orange to brown (Figure 3A). Deletion of the Fab-7 insulator resulted in a slight enhancement of pigmentation in 9 out of 14 transgenic lines, suggesting that Fab-7 functioned as a weak enhancer blocker in these transgenic lines. As in the case of transgenic lines carrying the *gypsy* insulator, the deletion of the eye enhancer and crossing into the *z^v77h^* mutant background reduced eye pigmentation in flies to the same extent (Figure 3A, S1B), suggesting a key role for Zeste in the eye enhancer activity and its ability to bypass the Fab-7 insulator.

Flies of the remaining 10 transgenic lines had yellow eyes. When the Fab-7 insulator was deleted, eye pigmentation was restored in only four lines (Figure 3B), suggesting that the eye enhancer in other six lines was inactive (data not shown). Thus, the Fab-7 insulator could effectively block the eye enhancer in only 4 out of 18 transgenic lines (22%) carrying the transgene with the functional eye enhancer.

We then examined eye pigmentation in transgenic lines homozygous for the construct (Figure 3C) in which the Fab-7 insulator displayed a weak enhancer-blocking activity. In all these lines, flies had darker eye color, compared to the lines heterozygous for the construct, suggesting that the Fab-7 insulator failed to effectively block the eye enhancer when the construct was in the homozygous state.

Taken together, these results indicate that the Fab-7 insulator can only weakly affect the activity of the eye enhancer in most of genomic positions. Both Fab-7 and *gypsy* insulators can effectively block the eye enhancer in approximately one-fourth of transgenic lines. In contrast to the *gypsy* insulator, the pairing of two Fab-7 insulators located on homologous chromosomes failed to improve the eye enhancer blocking.

### Role of pairing between two identical insulators, *gypsy* or Fab-7, flanking the eye enhancer in blocking its activity

**Figure 4:**
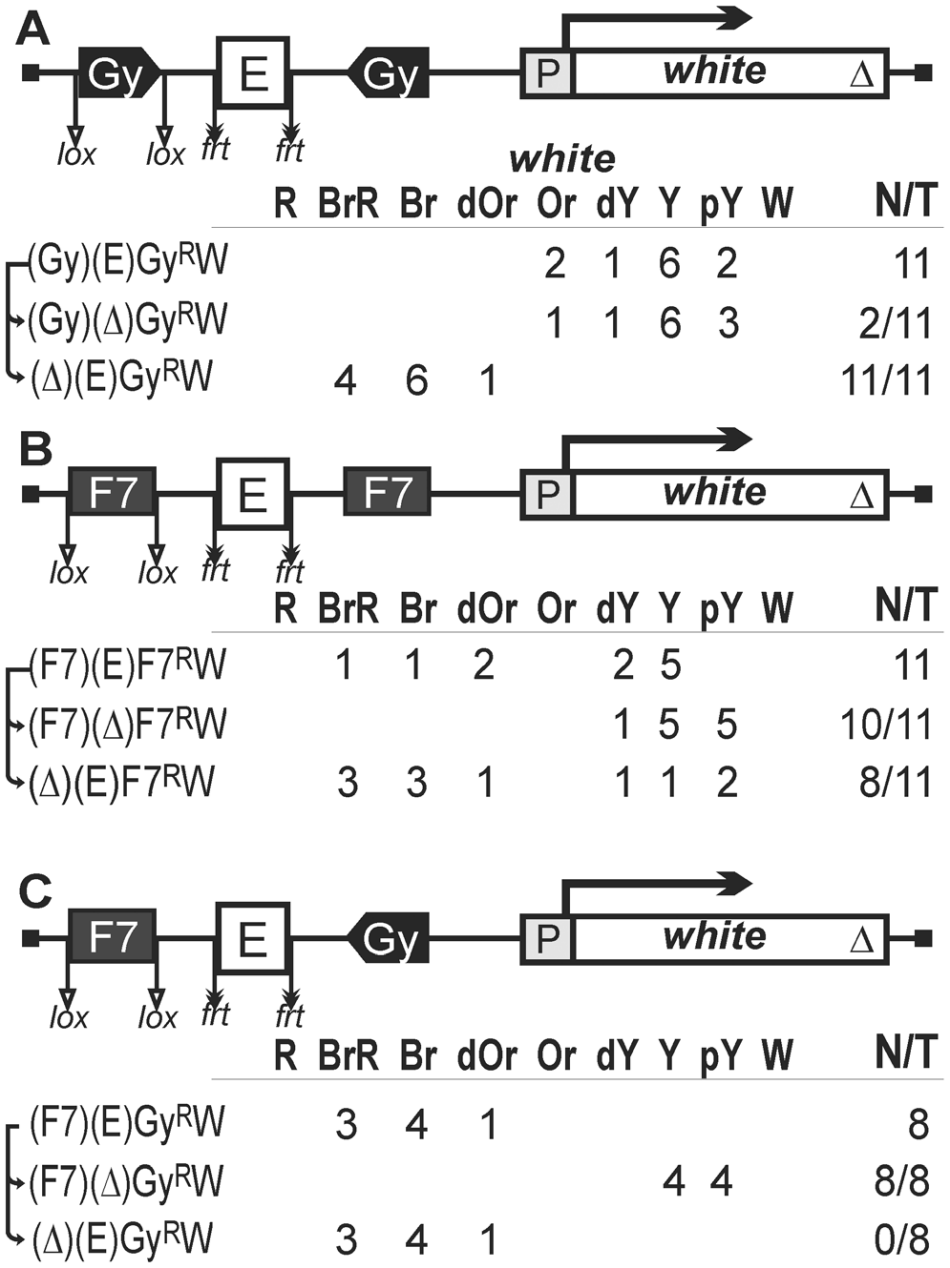
Testing functional interactions between *gypsy* and Fab-7 insulators. (A) Two *gypsy* insulators flank the eye enhancer. (B) Two Fab-7 insulators flank the eye enhancer. (C) One Fab-7 and one *gypsy* insulators flank the eye enhancer. Superscript “R” indicates that the corresponding element is inserted in the reverse orientation relative to the *white* gene in the construct. Other designations are as in Figure 1.

Since one copy of the *gypsy* or Fab-7 insulator in most insertion sites of the transgenes failed to block the eye enhancer, we decided to test if the eye enhancer placed between two insulators would improve the efficiency of blocking. For this purpose, we made constructs where the eye enhancer was flanked by a pair of *gypsy* insulators (Figure 4A) or Fab-7 insulators (Figure 4B) inserted in opposite orientations. In both cases, the insulator located upstream of the eye enhancer was flanked by lox sites, which allowed us to assess its role in blocking the eye enhancer.

In all 11 transgenic lines carrying the construct with two *gypsy* insulators (Figure 4A), flies had eye pigmentation in the range from pale yellow to orange, indicating that the eye enhancer activity was strongly suppressed. Deletion of the eye enhancer resulted in a slight reduction of pigmentation in only 2 out of 11 lines, providing evidence that the eye enhancer was strongly blocked in all these lines. Deletion of the upstream insulator restored eye pigmentation in 10 transgenic lines (Figure 4A), while subsequent deletion of the eye enhancer reduced it to the initial level (data not shown). These results showed that two copies of the *gypsy* insulator flanking the eye enhancer completely blocked its activity.

In transgenic lines carrying the construct with two Fab-7 insulators (Figure 4B), we observed a wide range of eye phenotypes, from brown-red to yellow. Deletion of the eye enhancer resulted in reduction of pigmentation in 10 out of 11 transgenic lines, showing that two Fab-7 insulators failed to effectively block the eye enhancer. However, the deletion of the upstream insulator provided for slight intensification of eye pigmentation in 8 out of 11 transgenic lines, suggesting that the interaction between the Fab-7 insulators could contribute to enhancer blocking.

To test whether two different insulators can cooperate in blocking the eye enhancer, we made the construct that contained one Fab-7 insulator flanked by lox sites inserted upstream of the eye enhancer and one *gypsy* insulator placed between the eye enhancer flanked by frt sites and the promoter (Figure 4C). We obtained eight transgenic lines that displayed high levels of eye pigmentation. Deletion of the eye enhancer strongly reduced eye pigmentation, while deletion of the Fab-7 insulator did not have any effect on eye color in any of the lines tested. This is evidence that the Fab-7 and *gypsy* insulators do not functionally interact in blocking the eye enhancer.

### Enhancer-blocking activity depends on the position of the eye enhancer in the insulator loop and on the relative orientation of interacting *gypsy* insulators

**Figure 5:**
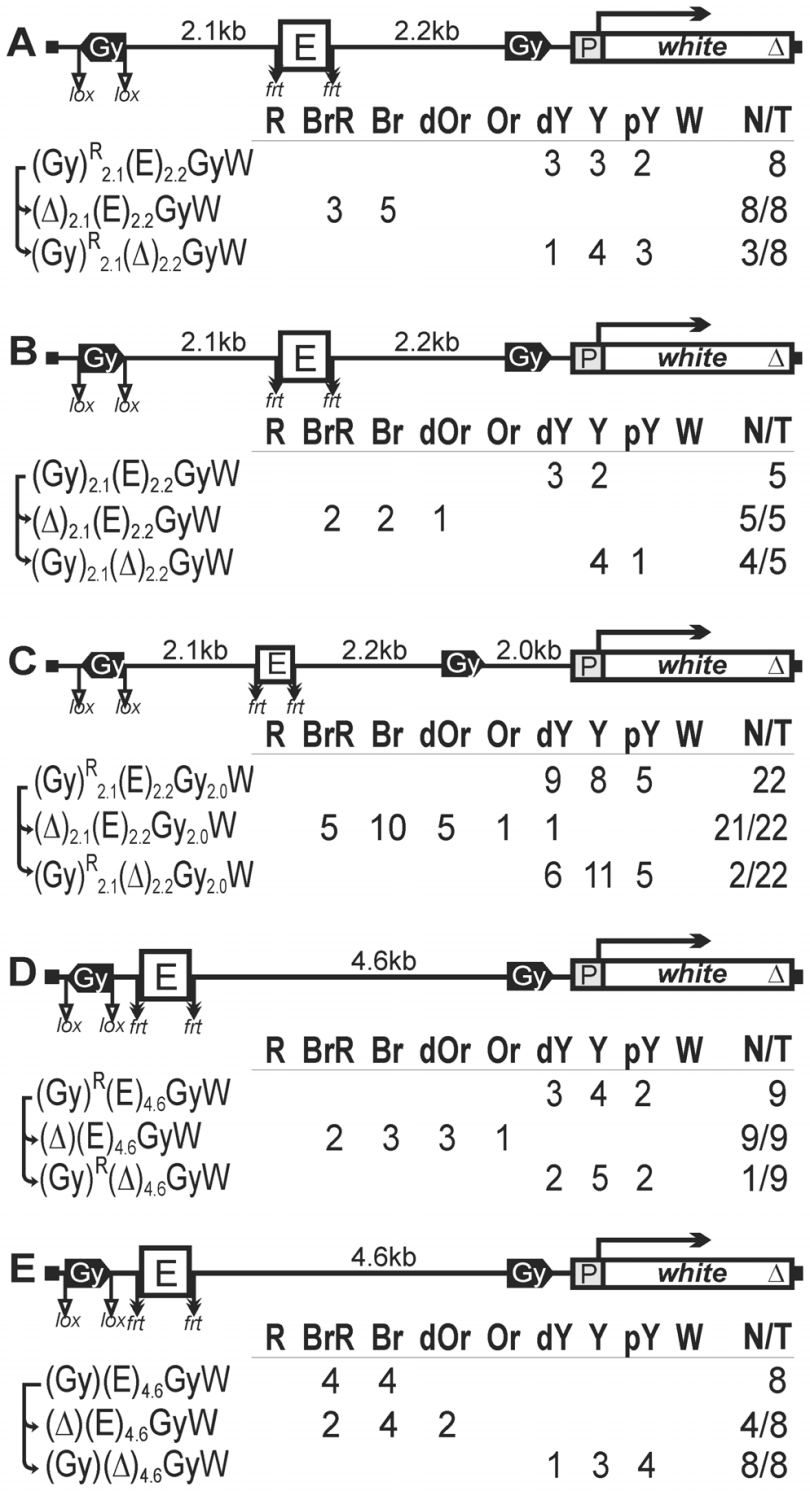
The role of *gypsy* orientation and distance between *gypsy* insulators in blocking the eye enhancer. The eye enhancer is inserted in the center of a 4.3-kb domain bordered by the *gypsy* insulators located (A) in the opposite or (B) in the same orientation. (C) Insertion of additional 2-kb DNA fragment between the 4.3-kb domain bordered by the *gypsy* insulators and the *white* promoter. (D, E) The eye enhancer is inserted in a 5.5-kb loop formed by the *gypsy* insulators located (D) in the opposite or (E) in the same orientation. Designations are as in Figures 1 and 4.

In transgenic lines described in Figure 4, the eye enhancer was tightly flanked by the *gypsy* insulators. Therefore, the putative insulator loop was probably quite small, and chromatin could be wound into a “tight knot” with consequent conformational and/or steric hindrances to the enhancer function. To test whether an increase in the distance between the *gypsy* insulators flanking the eye enhancer can restore enhancer–promoter communication at the *white* gene, we inserted the frt-flanked eye enhancer in the center of a 4.3-kb fragment bordered by the *gypsy* insulators in either the opposite (Figure 5A) or the same orientation (Figure 5B). The upstream *gypsy* insulator was flanked by lox sites.

In both series of transgenic lines, flies had eye pigmentation in a dark-yellow to pale-yellow range. The deletion of the eye enhancer slightly reduced eye pigmentation in 7 out of 13 transgenic lines. The deletion of the upstream *gypsy* insulator restored eye pigmentation in all 13 transgenic lines, indicating that one copy of the *gypsy* insulator failed to block the eye enhancer. These results showed that a 5.2-kb loop (4.3-kb DNA fragment and 0.9-kb frt-flanked eye enhancer) formed by the *gypsy* insulators allowed blocking of the eye enhancer located in the center of the loop. Importantly, the enhancer blocking did not depend on the relative orientation of the *gypsy* insulators.

In all the above constructs, the *white* promoter was located in close proximity to one of the insulators that formed the chromatin loop around the eye enhancer. In such configuration of the regulatory elements, the promoter might be unable to interact with the eye enhancer due to steric hindrances. To test for the role of distance between the *white* promoter and the insulator loop in this process, we modified the construct shown in Figure 5A by inserting an additional 2-kb DNA fragment between the *gypsy* insulator and the *white* promoter (Figure 5C). Once again, the interacting *gypsy* insulators effectively blocked the eye enhancer in most of transgenic lines, while one copy of the insulator had no enhancer-blocking activity.

In the next series of experiments, we tried to test whether the relative orientation of the *gypsy* insulators is important for the enhancer blocking when the eye enhancer is located in close proximity to the insulator inside the loop. For this purpose, we inserted the frt-flanked eye enhancer in close proximity to the upstream *gypsy* insulator. As a result, the eye enhancer was inside the 5.5-kb loop formed by the insulators located in either the opposite (Figure 5D) or the same orientation (Figure 5E).

In lines carrying the transgene with *gypsy* insulators in opposite orientations, flies had eye pigmentation ranging from dark yellow to pale yellow, which remained unchanged after the deletion of the eye enhancer. Thus, in such configuration of regulatory elements, *gypsy* insulators completely blocked the eye enhancer. The deletion of the proximal *gypsy* insulator restored the enhancer activity, suggesting that the interaction between the insulators was critical for blocking the eye enhancer. Interestingly, when the insulators were in the same orientation, flies had brown eyes and the deletion of the enhancer strongly reduced eye pigmentation, which was indicative of a role for the enhancer in stimulating of the *white* expression. The deletion of the proximal insulator also slightly reduced eye pigmentation in half of the transgenic lines. Thus, the *gypsy* insulators located in the same orientation allow the interaction between the eye enhancer located within the loop and the *white* promoter located outside the loop.

### Addition of the *gypsy* insulator downstream of the *white* gene improves the enhancer-blocking activity of the upstream insulator and potentiates promoter activity

**Figure 6:**
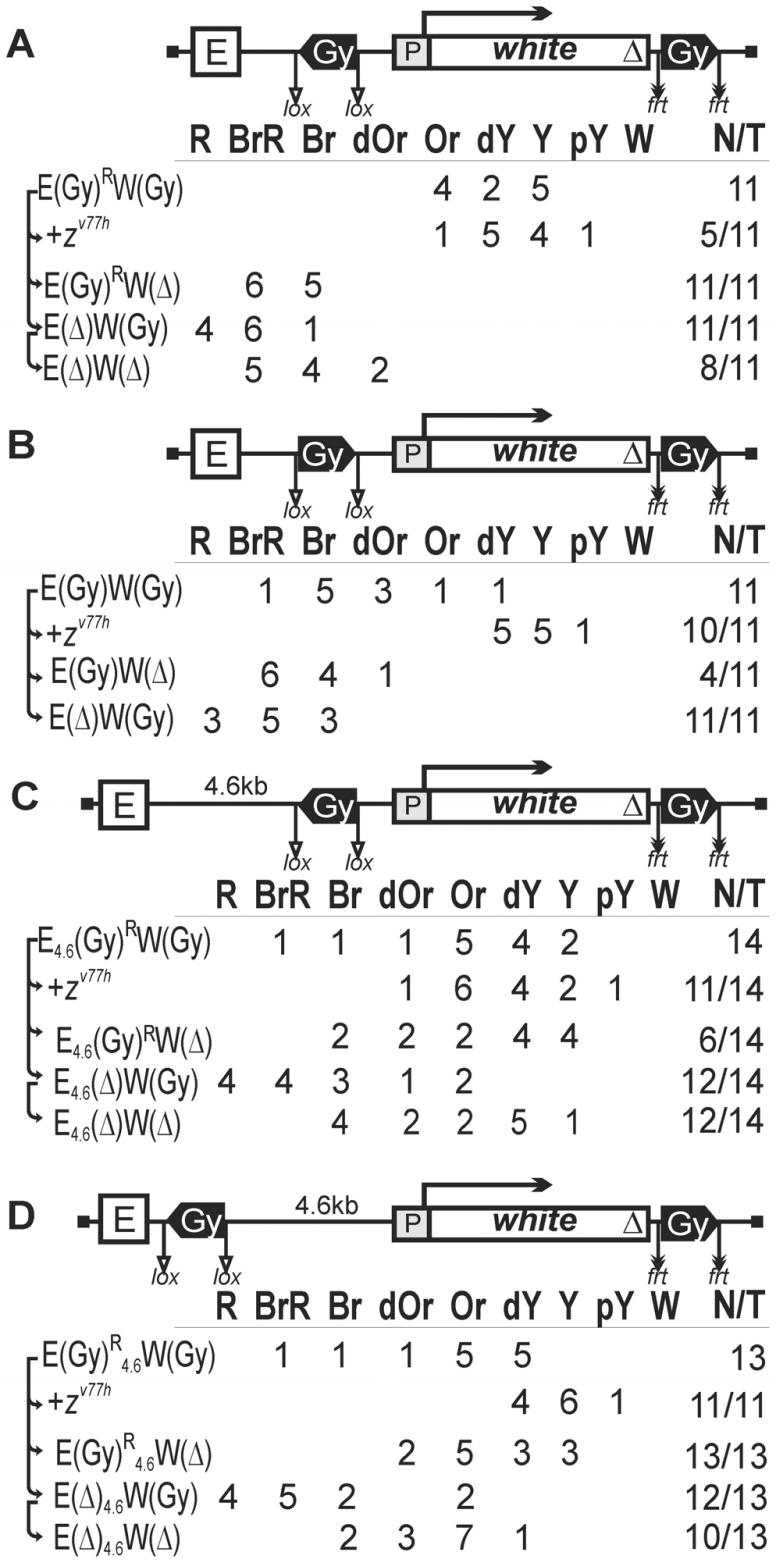
Testing the activities of *gypsy* insulators flanking the *white* gene. The *gypsy* insulators flanking the *white* gene are inserted either (A) in the opposite or (B) in the same orientation. (C) Insertion of the 4.6-kb DNA fragment between the eye enhancer and the *white* gene domain flanked by the *gypsy* insulators. (D) Insertion of the 4.6-kb DNA fragment between the *gypsy* insulator and the *white* promoter. Designations are as in Figures 1 and 4.

Next, we tested if placing *gypsy* insulators on both sides of the *white* gene would also lead to the improvement of enhancer-blocking activity. In two constructs, one frt-flanked *gypsy* insulator was inserted downstream of the *white* gene, and the other, lox-flanked *gypsy* insulator was inserted between the eye enhancer and the promoter in either the opposite (Figure 6A) or the same orientation (Figure 6B). As a result, the eye enhancer was located upstream of the chromatin domain formed by two *gypsy* insulators bordering the *white* gene.

When the *gypsy* insulators were in opposite orientations, the activity of the eye enhancer was almost completely blocked (Figure 6A): flies in 11 transgenic lines had eye pigmentation in the range from orange to yellow. As Zeste is essential for the eye enhancer activity, we regarded the eye phenotype in the *z^v77h^* background as resulting from deletion of the eye enhancer. Crossing transgenic lines into the mutant *z^v77h^* background only weakly diminished eye pigmentation in 5 out of 11 lines (Figure 6A), confirming that the eye enhancer was inactive. At the same time, the deletion of the downstream *gypsy* insulator restored *white* expression, suggesting that the interaction between the *gypsy* insulators is critical for enhancer blocking.

When the *gypsy* insulators were inserted in the same orientation (Figure 6B), flies displayed higher levels of eye pigmentation. Crossing the transgenes into the *z^v77h^* background significantly reduced eye pigmentation in all test lines, indicating that the eye enhancer was partially active. Therefore, when the *gypsy* insulators had the same orientation, their enhancer-blocking potential was reduced.

To test if the distance between the chromatin domain formed by insulators bordering the *white* gene and the eye enhancer is important for insulation, we inserted a 4.6-kb DNA fragment between the eye enhancer and the proximal *gypsy* insulator (Figure 6C). The *gypsy* insulators were placed in opposite orientations. In 14 transgenic lines, flies displayed a wide range of eye colors, from brown-red to yellow. The *z^v77h^* mutation reduced eye pigmentation in 11 out of 14 lines; i.e., the eye enhancer could stimulate transcription in most of the lines. Thus, an increase in the distance between the eye enhancer and the chromatin domain formed by the *gypsy* insulators diminished the insulating effect of the loop.

Next, we inserted the 4.6-kb fragment between the *gypsy* insulator and the *white* promoter (Figure 6D) so that the eye enhancer was near the upstream insulator. As a result, the *white* promoter was in the center of the chromatin domain formed by the *gypsy* insulators inserted in opposite orientations. Once again, we found that the eye enhancer was partially active in all transgenic lines, indicating that the distance between the enhancer or promoter and the *gypsy* insulator is important for blocking activity.

Comparisons of eye phenotypes in all derivative transgenic lines before and after deletion of the *gypsy* insulator located on the 3′ side of the *white* gene (Figures 6A, 6C, 6D) showed that the downstream *gypsy* insulator effectively stimulated *white* expression. Thus, the *gypsy* insulator placed at the end of the *white* gene can potentiate the *white* promoter activity.

### Mod(mdg4)-67.2 is required for blocking of the eye enhancer by paired *gypsy* insulators

**Figure 7:**
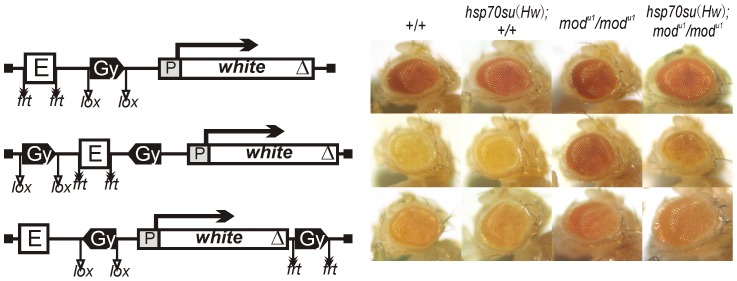
The role of Su(Hw) and Mod(mdg4)-67.2 proteins in the enhancer blocking by the *gypsy* insulator. Photographs show eye phenotypes in flies from the transgenic lines carrying either of the constructs in the wild-type (+/+) or the *mod(mdg4)^u1^* mutant (mod) background. Expression of Su(Hw) in transgenic lines carrying the *hsp70su(Hw)* construct was induced by heat shock. Other designations are as in Figure 1.

According to FlyBase data, the *su(Hw)* gene is weakly expressed in the eyes of adult flies. Therefore, the level of the Su(Hw) protein is also low, which may account for the inability of a single copy of the *gypsy* insulator to block the eye enhancer. To test this possibility, we produced three transgenic lines carrying the *su(Hw)* gene under control of the *hsp70* promoter (Figure S2A). The elevated level of Su(Hw) had no effect on eye pigmentation in transgenic lines with either one or two *gypsy* insulators (Figures 7, S3) suggesting that the concentration of this protein is not critical for eye enhancer blocking by the *gypsy* insulator. Further stimulation of Su(Hw) expression by heat shock (Figure 7, S3) also had no effect on eye pigmentation in any of transgenic lines. Thus, overexpression of Su(Hw) failed to provide for eye enhancer blocking by one copy of the *gypsy* insulator.

Next, we examined the role of Su(Hw) and Mod(mdg4)-67.2 proteins in blocking the eye enhancer by two copies of the *gypsy* insulator. To test Su(Hw), we used the *su(Hw)^v^/su(Hw)^2^* combination of mutations that significantly reduced the amount of the Su(Hw) protein [66], [67]. In the *su(Hw)^v^/su(Hw)^2^* background, eye pigmentation was restored to the same level as after deletion of the *gypsy* insulator, indicating that Su(Hw) is critical for insulation (data not shown).

In the *mod(mdg4)^u1^* and *mod(mdg4)^T6^* mutations, the truncated Mod(mdg4)-67.2 with deleted C-terminal domain partially lost its functional activity [47], [68]. Both mutations significantly but not completely restored eye pigmentation in the transgenic lines carrying two copies of the *gypsy* insulator (Figures 7, S3), suggesting a role for Mod(mdg4)-67.2 in blocking the eye enhancer.

To further test the role of the insulator proteins, we examined the effect of the *mod(mdg4)^u1^* mutation in combination with overexpression of the Su(Hw) protein (Figure 7, S3). The *hsp70su(Hw)* transgene did not affect *white* expression in the *mod(mgd4)* mutant background in any of transgenic lines, suggesting that a moderate increase in the amount of Su(Hw) is insufficient for counterbalancing Mod(mdg4)-67.2 inactivation. However, strong overexpression of Su(Hw) after induction by heat shock proved to partially restore enhancer blocking by paired *gypsy* insulators suppressed by the *mod(mdg4)^u1^* and *mod(mdg4)^T6^* mutations (Figure 7, S3).

Previously it was found that, in the *mod(mdg4)* mutant background, the *gypsy* insulator directly repressed the *yellow* promoter in pupae [68] and the *white* promoter in embryos [69]. Therefore, overexpression of Su(Hw) in the *mod(mdg4)* mutant background could possibly lead to direct repression of the *white* promoter. However, induction of Su(Hw) expression by heat shock had no effect on eye pigmentation in flies carrying one copy of the *gypsy* insulator in the *mod(mdg4)* mutant background (Figure 7). Thus, a high level of Su(Hw) did not induce direct repression of the *white* promoter. These results suggest that Mod(mdg4)-67.2 is required for blocking the eye enhancer by paired *gypsy* insulator and that overexpression of Su(Hw) can partially compensate for inactivation of Mod(mdg4)-67.2 in the *mod(mdg4)* mutations.

Interestingly, a similar result was obtained with the *ct^6^* mutation, a classical model for testing the activity of the *gypsy* insulator. In the *ct^6^* allele, a *gypsy* element is inserted close to and completely blocks a wing margin enhancer located about 85 kb upstream of the *cut* promoter [47], producing a cut wing phenotype (Figure S2B). The *mod*(*mdg4*)*^u1^* mutation almost completely suppressed the *ct^6^* mutant phenotype, suggesting that Mod(mdg4)-67.2 is essential for blocking the wing enhancer. Overexpression of Su(Hw) in the *mod(mdg4)* mutant background partially rescued the blocking of the wing enhancer, resulting in an intermediate cut wing phenotype. Thus, a high level of Su(Hw) can partially counterbalance the negative influence of the *mod(mdg4)^u1^* mutation on insulation. This is also supported by the previous observation that overexpression of Su(Hw) partially restored *y^2^* expression in bristles that was repressed in the *mod(mdg4)^u1^* background [70].

Finally, we tested whether Mod(mdg)-67.2 is essential for the ability of the distal *gypsy* insulator located on the 3′ side of the gene to stimulate *white* expression. We examined six transgenic lines (described in Figure 6) carrying only the distal *gypsy* insulator and the eye enhancer located either close to the *white* promoter (Figure S4 A) or at a distance of 4 kb from it (Figure S4 B, C). The results showed that deletion of the *gypsy* insulator reduced *white* expression in all cases, while its reduction in *mod(mdg4)^u1^* mutants was relatively weak. Thus, Mod(mdg4)-67.2 proved to be essential but not critical for the ability of the *gypsy* insulator to stimulate *white* expression from a distance.

### The *gypsy* insulator directly interacts with the eye enhancer *in vivo*

**Figure 8:**
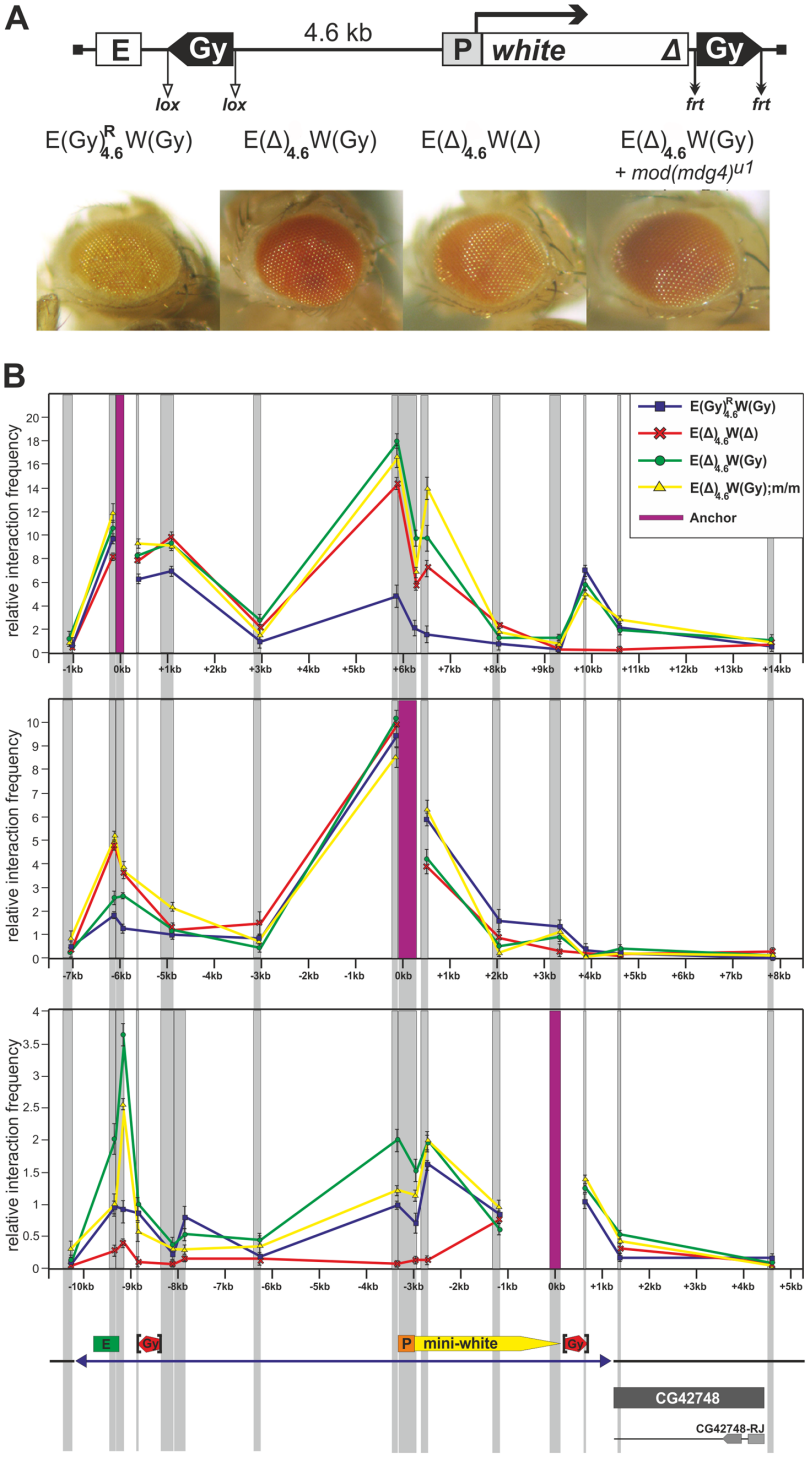
The *gypsy* insulators directly interact with the enhancer and promoter of the *white* gene. (A) Eye phenotypes in flies from the transgenic line heterozygous (P/+) for the construct and in flies after deletion of either the proximal *gypsy* insulator, or both insulators, or introduction of the *mod(mdg4)^u1^* mutation. (B) The 3C profile of the E(Gy)^R^_4.5_W(Gy) line and its derivatives analyzed at the pupa stage for an anchor fragment located in the enhancer or promoter region or downstream of the distal *gypsy* insulator. The transgene and the surrounding genomic region are drawn to scale. Relative interaction frequencies between *Dpn*II restriction fragments selected for this analysis (gray vertical bands) and anchor regions (purple vertical bands) are shown for the construct (blue-square line), and its derivatives with deletion of proximal *gypsy* (green-circle line), proximal *gypsy* at the *mod(mdg4)^u1^* background (yellow-triangle line), and both *gypsy* copies (red-cross line). Samples were normalized by qPCR relative to an undigested locus. Error bars show standard deviations from the means of three independent experiments. Transgene DNA is shown as blue line at the bottom of figure, and the boundaries of the transgenes are indicated by blue triangles. The *white* gene, eye enhancer, and *gypsy* insulator are represented, respectively, by yellow and green boxes and red pentagon. The lox and frt sites allowing the excision of insulators are indicated by vertical black brackets. Other designations are as in Figure 1.

The ability of the *gypsy* insulator located on the 3′ side of the *white* gene to stimulate its expression suggested that the insulator may directly interact with the *white* regulatory regions. To test this possibility, we used the 3C assay to examine long-distance interactions in pupae from a transgenic line homozygous for the construct shown in Figure 6D. In this transgene (Figure 8A), the eye enhancer was isolated by the proximal *gypsy* insulator located at 4.6 kb from the *white* promoter, while the distal *gypsy* insulator was inserted downstream of the *white* gene. We examined the original transgenic line and two derivatives carrying either the distal *gypsy* insulator or no insulators. We also tested the role of the *mod(mdg4)^u1^* mutation in the interaction between the distal *gypsy* insulator and the regulatory regions of the *white* gene.

Using anchors at the eye enhancer, the *white* promoter, and the distal *gypsy* insulator, we observed strong interaction between the *gypsy* insulators (Figure 8B). The interaction between the eye enhancer and the promoter was reduced, correlating with the low level of *white* expression in the transgenic line carrying two copies of the *gypsy* insulator. After deletion of the proximal *gypsy* copy, the enhancer-promoter interaction was considerably increased, and this was accompanied by stimulation of *white* expression by the enhancer in the derivative transgenic line. Thus, the interaction between two *gypsy* insulators partially blocked the eye enhancer.

When the distal insulator alone was present in the transgene, this insulator interacted with the promoter and the eye enhancer. The *mod(mdg4)^u1^* mutation did not significantly affect these interactions. In the absence of distal *gypsy* insulators, no interactions were found between the regulatory regions and the 3′ side of the *white* gene, confirming that the *gypsy* insulator is essential for the observed contacts. In correlation with the observed interactions, deletion of the distal insulator, but not introduction of the *mod(mdg4)^u1^* mutation, strongly reduced *white* expression. These results support the model that the *gypsy* insulator directly interacts with the *white* enhancer/promoter and stimulates its basal activity.

**Figure 9:**
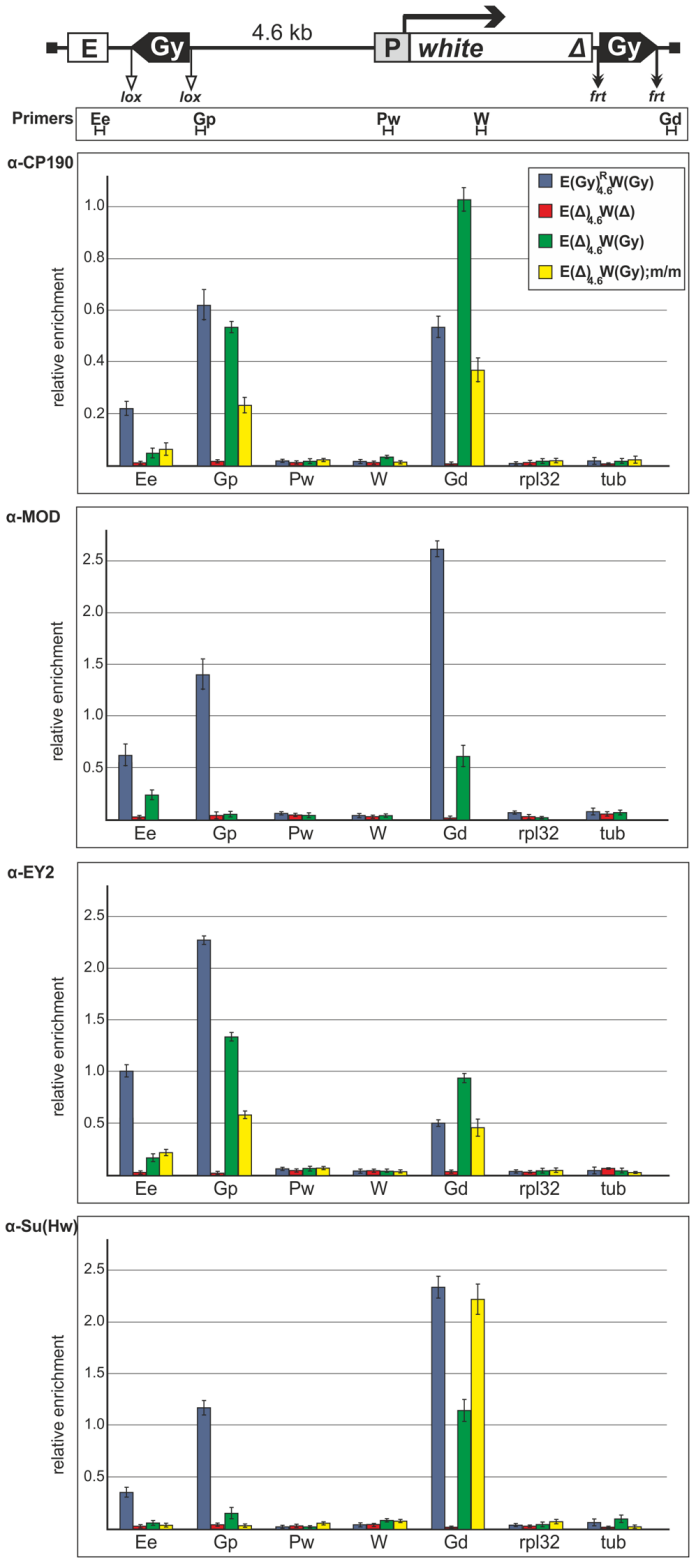
Binding of insulator proteins to the eye enhancer, promoter, *white* gene and *gypsy* insulators in the transgenic construct. Chromatin was isolated from transgenic flies carrying the construct or its derivatives described in Figure 8 was treated with antibodies against CP190, Mod(mdg4)-67.2 (designated as MOD), E(y)2, and Su(Hw). The results of ChIP are presented as a percentage of input DNA normalized relative to the endogenous positive binding site for insulator proteins from the 62D region. Relative locations of primers for ChIP are indicated under the construct scheme. Proximal and distal *gypsy* insulators are designated as Gp and Gd correspondingly. The *rpl32* and *tubulin* (tub) coding regions were used as controls devoid of binding sites for the test proteins. Error bars indicate standard deviations of quadruplicate PCR measurements. Other designations are as in Figure 1.

To further verify the ability of the *gypsy* insulator to interact with the *white* regulatory elements, we used chromatin immunoprecipitation (ChIP) assay to analyze binding of the insulator proteins Su(Hw), E(y)2, CP190, and Mod(mdg4)-67.2 to several sites in the promoter, enhancer and insulator regions in pupae from the same transgenic line and its derivatives (Figure 9). In the derivative transgenic line carrying the construct without *gypsy* insulators, the insulator proteins were not detected on the enhancer and promoter of the *white* gene. The recruitment of these proteins to the transgene only by the *gypsy* insulator allowed us to test interaction of the *gypsy* insulator with the *white* regulatory regions. It could be expected that the insulator proteins would be detected on the enhancer or/and promoter if these regulatory elements directly interacted with the insulator. The insulator proteins proved to bind to the insulators and the eye enhancer in the transgenic line carrying two copies of the *gypsy* insulator, but no binding took place after both copies were deleted. Therefore, pairing of the *gypsy* insulators did not preclude their interaction with the eye enhancer. When the proximal insulator alone was deleted, we still detected the insulator proteins binding to the eye enhancer (Figure 9). Taken together, these results strongly suggest that one or two copies of the *gypsy* insulator can directly interact with the eye enhancer.

In the *mod(mdg4)^u1^* background (Figure 9), the E(y)2 and CP190 proteins continued to bind to the eye enhancer region, confirming the 3C results that the *gypsy* insulator interacts with the eye enhancer in the absence of Mod(mdg4)-67.2 protein. This finding correlates with data on the ability of the *gypsy* insulator to stimulate transcription in the *mod(mdg4)^u1^* background.

### Interaction of Mod(mdg4)-67.2 with Zeste may be involved in blocking of the eye enhancer

As shown previously, Zeste has binding sites in the enhancer and promoter regions of the *white* gene [64], and these sites in the promoter region are essential for the insulator bypass by the eye enhancer [71].

**Figure 10:**
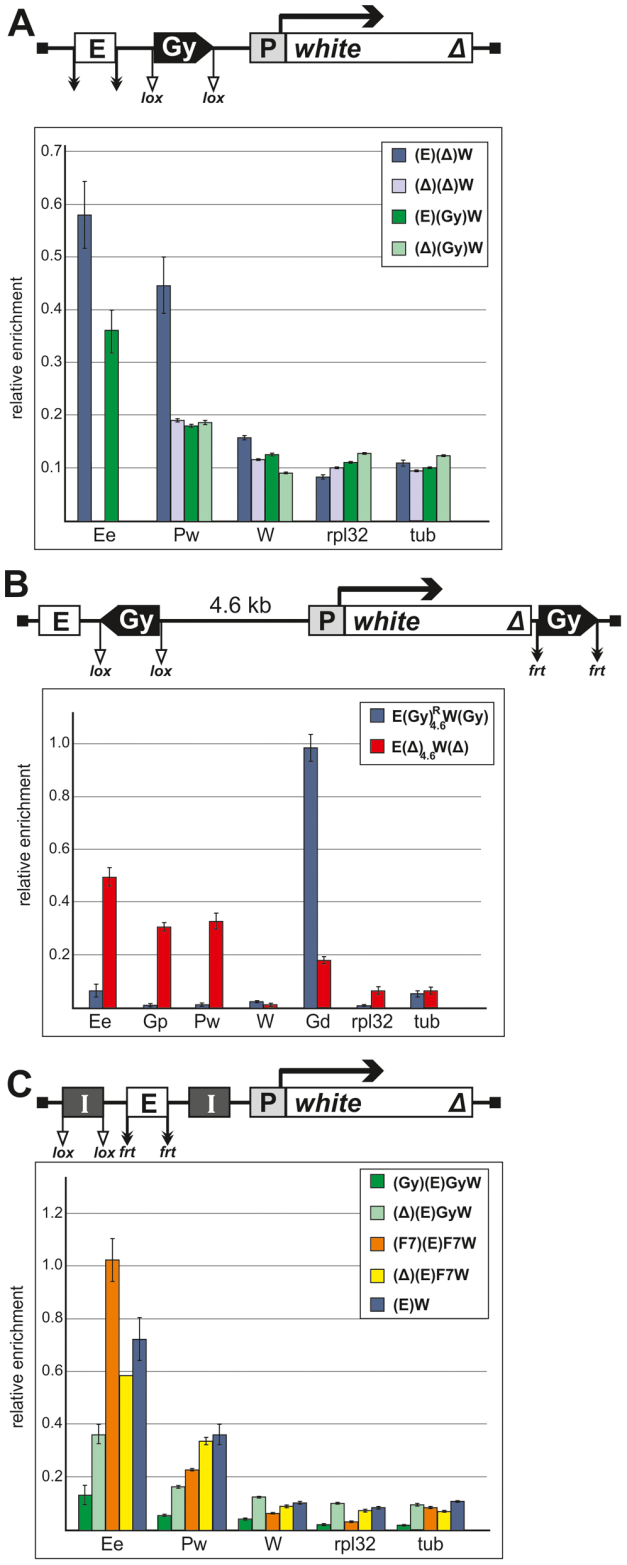
Binding of Zeste to the *white* enhancer, promoter, and coding region in the transgenic constructs. Results of immunoprecipitation experiments with chromatin isolated from transgenic flies and treated with anti-Zeste antibodies. (A) The results of ChIP (percentages of input DNA normalized relative to the endogenous positive binding site for Zeste from the *Ubx* promoter region) of specified chromatin regions with antibodies to Zeste in the transgenic construct in the presence or absence of *gypsy* insulator (Gy) in one copy. (B) The results of ChIP with antibodies to Zeste in the derivatives of the transgenic construct described in Figure 8 in the presence or absence of the *gypsy* insulator (Gy) in one copy. (C) Comparison of the results of ChIP with anti-Zeste antibodies in the presence of two copies of either *gypsy* or Fab-7 insulators (I, black boxes). Designations: E (the eye enhancer), P (promoter) and W (coding region) of the *white* gene. The *rpl32* and *tubulin* (tub) coding regions were used as controls devoid of Zeste binding sites. Error bars indicate standard deviations of quadruplicate PCR measurements. Other designations are as in Figure 1 and 9.

Using the ChIP assay, we analyzed the binding of Zeste to the eye enhancer and the promoter of the *white* gene in pupae from the transgenic line homozygous for the construct and compared its binding to the *white* promoter before and after deletion of the eye enhancer (Figure 10A). As expected, Zeste bound to the eye enhancer and the *white* promoter in the initial line, but only traces of this protein were detected on the *white* promoter in the derivative transgenic line with the deleted eye enhancer. Thus, Zeste was found to be recruited to the *white* promoter only in the presence of the eye enhancer. In agreement with this finding, inactivation of Zeste in the *z^v77h^* mutants did not affect *white* expression in absence of the eye enhancer (Figure S1). Zeste was not detected on the *white* promoter in the transgenic line carrying the *gypsy* insulator between the eye enhancer and the promoter, in agreement with data on the blocking of the eye enhancer by one copy of the *gypsy* insulator in transgenic lines homozygous for the construct (Figure 10A).

Next, we examined the binding of Zeste to the transgenic line and its derivatives described in Figures 8 and 9. In the presence of two *gypsy* insulators, the enrichment of enhancer and promoter sequences upon ChIP with anti-Zeste antibodies was considerably reduced (Figure 10B). However, we unexpectedly observed strong Zeste binding to the distal insulator. Similar results were obtained with two additional transgenic lines in which the *white* gene was flanked by the *gypsy* insulators (Figure S5). For both transgenic lines, we observed strong enrichment of sequences related to the distal *gypsy* insulator upon ChIP with anti-Zeste antibodies. At the same time, binding of Zeste to the promoter and the enhancer was considerably reduced.

After deletion of the proximal *gypsy* insulator (Figure 10B), the level of Zeste at the *white* enhancer and promoter was still low, but this protein was detected at the reference sequence near the eye enhancer. After deletion of both *gypsy* insulator, Zeste was again detected by ChIP on the eye enhancer and the promoter. To confirm these results, we analyzed the enrichment of the enhancer and promoter regions by ChIP with anti-Zeste antibodies in several additional transgenic lines. The enrichment of enhancer sequences was considerably reduced in all transgenic lines carrying one or two copies of the *gypsy* insulator near the eye enhancer (Figures 10A, 10B, S5), but it returned to the reference level after the insulators were deleted.

In experiments with pupae carrying the transgene with the eye enhancer flanked by two Fab-7 insulators, the enrichment of the enhancer sequences upon ChIP with anti-Zeste antibodies was even higher than in experiments with the control transgenic line carrying only the eye enhancer or the derivative transgenic line with the deleted upstream Fab-7 insulator (Figure 10C). Thus, proteins bound to the Fab-7 insulator did not interfere with Zeste.

The plausible explanation of these results is that the proteins bound to the *gypsy* insulators interacted with Zeste and partially masked the epitopes recognized by the antibodies in ChIP. As a result, Zeste was not detected at the eye enhancer and promoter but was found at the distal *gypsy* insulator.

**Figure 11:**
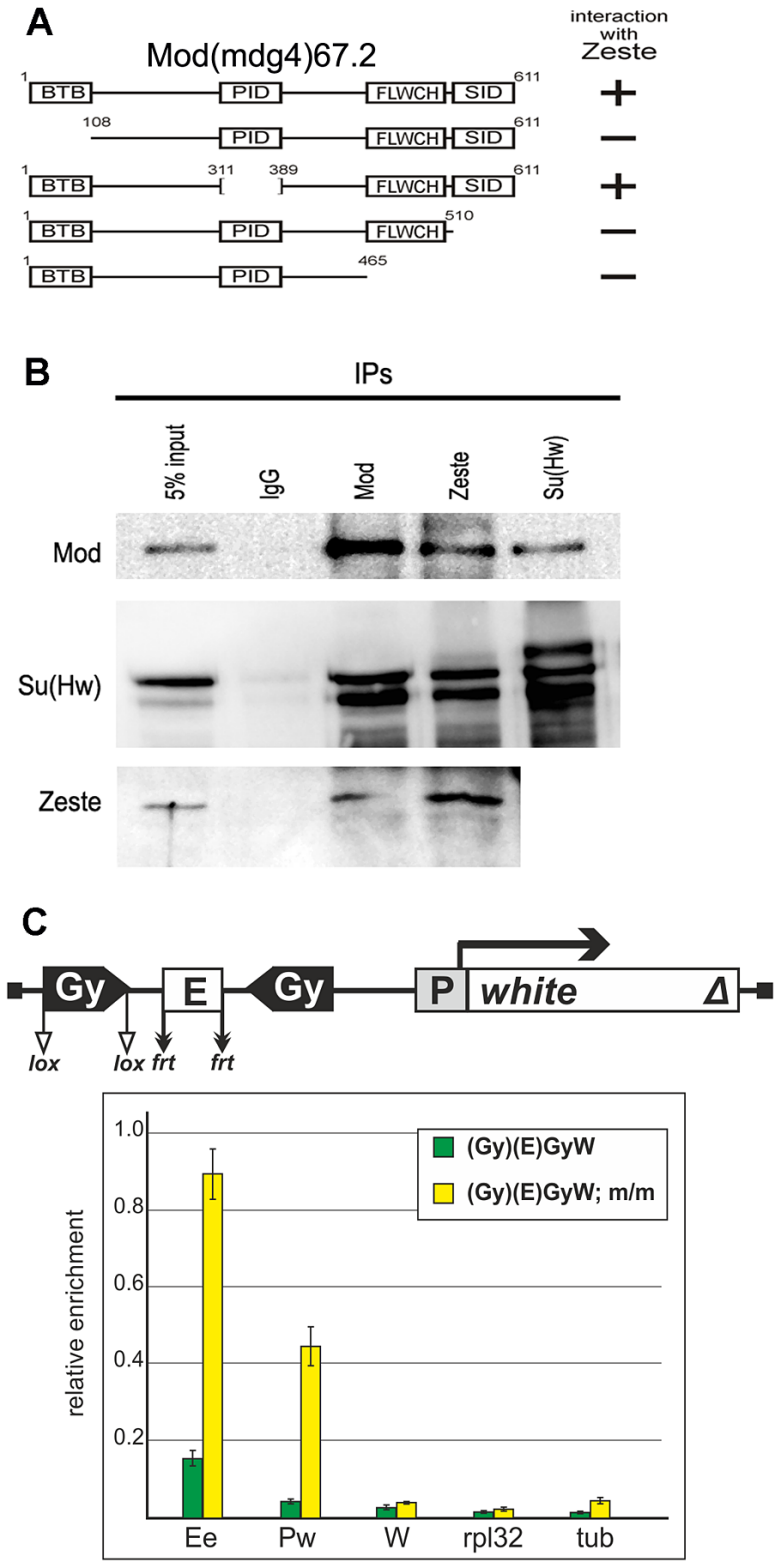
Testing for the interaction between the Mod(mdg4)-67.2 and Zeste proteins. (A) Summary of interactions between Mod(mdg4)-67.2 and Zeste in the yeast two-hybrid assay. Schemes show the structure of the full-length Mod(mdg4)-67.2 and its deletion derivatives, indicating the main domains of this protein. Plus and minus signs refer to a relatively strong interaction and the absence of interaction, respectively. Different fragments of Mod(mdg4)-67.2 were individually fused to the C-terminus of the GAL4 activating domain and tested for the interaction with Zeste fused to the C terminus of GAL4 DNA binding domain. All Mod(mdg4) fragments were tested for the absence of interaction with GAL4 DNA binding domain alone. (B) Nuclear extracts from *Drosophila* S2 cells were immunoprecipitated with antibodies specific for the indicated proteins (with preimmune IgG used as a negative control), and the immunoprecipitates (IPs) were analyzed by Western blotting for the presence of Mod(mdg4)-67.2 (designated Mod), Su(Hw) and Zeste proteins. (C) The results of ChIP (percentage of input DNA normalized relative to the endogenous positive binding site for Zeste from the *Ubx* promoter region) of specified chromatin regions with antibodies to Zeste in the transgenic construct. Analysis was performed with wild-type and *mod(mdg4)^u1^/mod(mdg4)^u1^* (m/m) pupae carrying the transgenic construct. Error bars indicate standard deviations of quadruplicate PCR measurements. Other designations are as in Figure 1 and 9.

To test this possibility, we examined the interaction of insulator proteins with Zeste in the yeast two-hybrid assay (Figure 11A). The results showed that Zeste interacted with Mod(mdg4)-67.2 but not with the Su(Hw) and CP190 proteins. It is noteworthy that both BTB and C-terminal domains of Mod(mdg4)-67.2 are essential for the interaction with Zeste. We also demonstrated co-immunoprecipitation between Zeste and Mod(mgd4)-67.2 in embryonic extracts (Figure 11B).

To confirm the role of Mod(mdg4)-67.2 in the interaction with Zeste, we performed ChIP experiments with pupae from two transgenic lines carrying a pair of *gypsy* insulators inserted on both sides of the eye enhancer in the *mod(mdg4)^u1^* mutant background (Figure 11C). In both transgenic lines, partial inactivation of Mod(mdg4)-67.2 restored enrichment with the eye enhancer sequence upon ChIP with the anti-Zeste antibodies. At the same time, the *mod(mdg4)^u1^* mutation did not affect Zeste expression in pupae (Figure S6). Taken together these results confirm the role of Mod(mdg4)-67.2–Zeste interaction in pairing of the eye enhancer with the *gypsy* insulator.

Finally, we used ChIP with anti-Zeste antibodies to test whether Mod(mdg4)-67.2 affected Zeste binding to the eye enhancer in transgenic lines carrying the *gypsy* insulator on the 3′ side of the *white* gene. The eye enhancer in these lines was located either close to the *white* promoter (Figure S7A) or 4.6 kb upstream of it (Figure S7B,C). We unexpectedly observed only a relatively weak enrichment of the enhancer sequence in the presence of the *gypsy* insulator, but its deletion or introduction of the *mod(mdg4)^u1^* mutation restored the normal level its enrichment in all transgenic lines. These results provide additional evidence for the direct interaction between Mod(mdg4)-67.2 and Zeste and the long-distance interaction of the insulator complex formed on *gypsy* sequences with the regulatory elements of the *white* gene.

## Discussion

In this study, we have examined two *Drosophila* insulators in the model system containing the eye enhancer and the *white* reporter gene lacking the endogenous Wari insulator that improves the enhancer-blocking activity of the *gypsy* insulator [10]. The results show that one copy of the *gypsy* or Fab-7 insulator fails to disrupt communication between the eye enhancer and the *white* promoter in the major part of transgenic lines, with the eye enhancer blocking being effective in only 22–28% of these lines. A plausible explanation to such a position-dependent enhancer blocking activity of the insulators is that there is only a minor part of genomic sites where an endogenous insulator and a transgenic insulator can form a loop that results in isolation of the eye enhancer. Alternatively, the strength of insulation depends on the functional activity of the eye enhancer, which depends on the site of transgene insertion [71].

**Figure 12:**
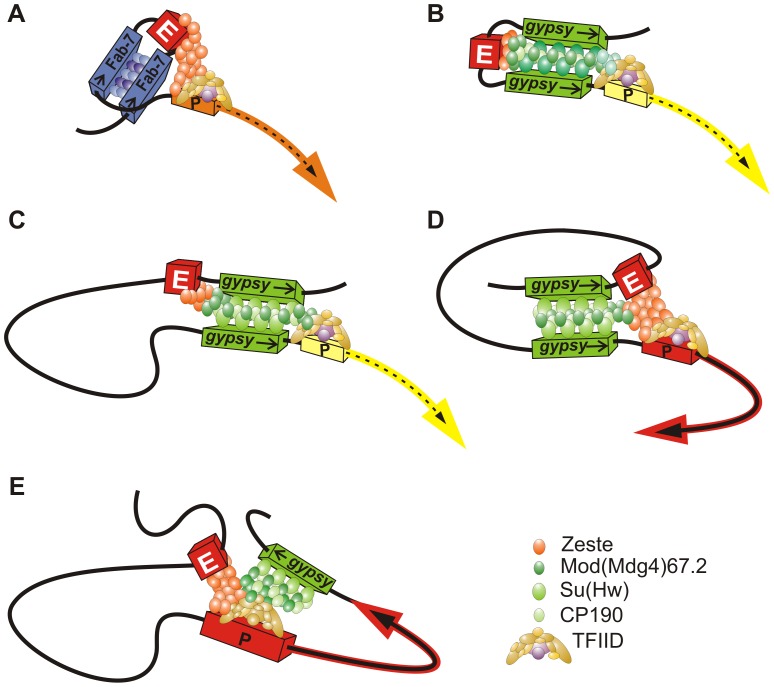
Model describing the mechanisms of enhancer blocking by the *gypsy* insulator. (A) The chromatin loop formed by the interacting insulators is not sufficient for effective blocking of the enhancer–promoter communication coordinated by Zeste. (B) The *gypsy* insulators block the eye enhancer by two different mechanisms: the chromatin loop formed by interacting *gypsy* insulators induces physical constrains on the enhancer–promoter communication; the interaction of Mod(mdg4)-67.2 with Zeste interferes with the enhancer-promoter communication. (C) The *gypsy* insulators located in opposite orientation block interaction between the eye enhancer located within the loop and *white* promoter located outside the loop; (D) The *gypsy* insulators located in the same orientation bring together the eye enhancer located within the loop and the *white* promoter located outside the loop. (E) The *gypsy* insulator located on the 3′ side of the *white* gene interacts with the enhancer and promoter.

We demonstrated the role of a putative chromatin loop formed by the *gypsy* insulators in blocking the eye enhancer. In general, two *gypsy* insulators flanking either the eye enhancer or the *white* gene effectively block the enhancer–promoter communication. However, two Fab-7 insulators fail to effectively block the eye enhancer activity, which is unlikely to be explained by their inability to form a loop around the eye enhancer. As shown previously, the interaction between the Fab-7 insulators can support long-distance enhancer–promoter communication [59] and higher-order organization of PcG targets in the nucleus [12]. Thus, the Fab-7 insulators should form a chromatin loop including the eye enhancer that fails to block the enhancer–promoter communication. Taken together, these results suggest that the chromatin loop formed by the interacting insulators is not sufficient for blocking enhancer–promoter communication coordinated by Zeste (Figure 12A). It appears that insulator complexes do not function as a neutral barrier and that specific interactions between insulator proteins and proteins bound either to an enhancer or to a promoter are essential for the effectiveness of blocking.

Here we have found that the proteins bound to the *gypsy* insulator effectively interact with the enhancer of the *white* gene. The *gypsy* insulator recruits Mod(mdg4)-67.2 that directly interacts with the Zeste protein. It appears that the BTB and C-terminal domains of Mod(mdg4)-67.2 are required for interaction with Zeste. Mod(mdg4)-67.2 can oligomerize through its BTB and second dimerization domain [51], [52], and this can help it to effectively interfere with the activity of Zeste, which also forms oligomers. Zeste is critical for the long-distance interaction between the *white* promoter and the eye enhancer [64], [71]. Thus, Mod(mdg4)-67.2 may interfere with the ability of Zeste to bring together remote regulatory elements. As the inactivation of Mod(mdg4)-67.2 does not completely disrupt the enhancer blocking, it seems likely that other insulator proteins also contribute to interactions with proteins bound to the eye enhancer and, therefore, may interfere with the enhancer–promoter communication.

The results presented above provide the basis for the model that two different mechanisms are involved in blocking the eye enhancer by the *gypsy* insulators: (1) a chromatin loop physically interferes with the ability of the protein complexes bound to the eye enhancer and promoter to interact with each other, and (2) direct interactions between insulator proteins and enhancer/promoter proteins interfere with the ability of an enhancer to properly stimulate a promoter. In particular, Mod(mdg4)-67.2 partially blocks the activity of Zeste via a direct protein–protein interaction (Figure 12B). These mechanisms function cooperatively, which ensures a strong blocking of the eye enhancer by the paired *gypsy* insulators. It seems likely that the protein complex bound to the Fab-7 insulator does not interfere with the activity of Zeste. As a result, a loop formed by the Fab-7 insulators only weakly affects communication between the eye enhancer and the *white* promoter.

As in the case of the insulator-mediated chromatin loop, the pairing of the *gypsy* insulators located on the homologous chromosomes may physically interfere with the enhancer–promoter communication. Thus, homologous pairing might be one of possible mechanisms contributing to the enhancer blocking activity of insulators. Supposedly, such mechanism may account for dosage compensation of some X-chromosomal genes that contain insulators between enhancers and promoters. Since paring between insulators can occur only in females, which have two X chromosomes, such insulators should block enhancers more effectively in females than in males.

Previously, we have used the Flp-recombination assay [53] to demonstrate that the pairing between *gypsy* insulators strongly depends on their relative orientation. According to our model, the orientation-dependent effect is explained by the involvement of at least two insulator-bound proteins in specific protein-protein interactions. Here we have found that when the eye enhancer is in the center of the loop, the relative orientation of the *gypsy* insulators is not critical for the efficient blocking of the eye enhancer. The opposite situation is observed when the eye enhancer is in close proximity to the upstream *gypsy* insulator (Figure 12C,D). In this configuration of the regulatory elements, the *gypsy* insulators located in the same orientation bring together the eye enhancer located within the loop and the *white* promoter located outside the loop. Therefore, the position of an enhancer relative to *gypsy* insulators within the loop appears to be critical for the functional outcome of loop formation.

The results of our study also support the model that insulators specifically interact with enhancers and promoters and potentiate their activity [72]–[74]. As shown previously, the endogenous Su(Hw)-binding region (1A2) placed at the 3′ end of the *white* gene in the transgenic line can interact with the promoter and stimulate its activity [74]. Here, we have found that the *gypsy* insulator located on the 3′ side of the *white* gene stimulates *white* expression by interacting with the enhancer (Figure 12D). The observed interaction between Mod(mdg4)-67.2 and Zeste is important but not critical for distant pairing of the eye enhancer and the *gypsy* insulator. Further study is required to identify additional transcription factors bound to the insulators, enhancers, and promoters that are involved in such interactions.

## Materials and Methods

### *Drosophila* strains, germline transformation, and genetic crosses

Flies were maintained at 25°C on standard medium. The construct, together with a P element containing defective inverted repeats (P25.7wc) that was used as a transposase source, were injected into *yacw^1118^* preblastoderm embryos [75]. The resulting flies were crossed with *yacw^1118^* flies, and the transgenic progeny were identified by their eye pigmentation under a Stemi 2000 stereomicroscope (Carl Zeiss, Germany). The transformed lines were tested for transposon integrity and copy number by Southern blot hybridization. Only single-copy transformants were included in the study.

The lines with DNA fragment excisions were obtained by crossing the transposon-bearing flies with the Flp (*w^1118^; S2CyO, hsFLP, ISA/Sco; +*) or Cre (*y^1^w^1^; Cyo, P[w+, cre]/Sco; +*) recombinase-expressing lines [76], [77]. The Cre recombinase induces 100% excisions in the next generation. A high level of Flp recombinase was produced by heat shock treatment (2 hours daily) during the first 3 days after hatching. All excisions were confirmed by PCR analysis.

To inactivate the Zeste protein, we used the null *z^v77h^* mutation (*z^v77h^ w^67c23^*, Bloomington Stock Center #1385), which contains a 314-bp deletion including RNA leader sequences and the AUG initiation codon of *zeste* [65]. To inactivate Mod(mdg4)-67.2, we used the *mod(mdg4)^u1^* and *mod(mdg4)^T6^* mutations associated with the deletion of the C-terminal protein domain interacting with Su(Hw) [48], [68].

Generation of transgenic lines and introduction of *z^v77h^, mod(mdg4)* mutations, *su(Hw)^2^/su(Hw)^v^* mutations, and *hsp70su(Hw)* construct were described previously [51], [68], [71]. To express the *su(Hw)* gene regulated by the *hsp70* promoter, flies carrying the construct were heat-shocked for 2 h every day during the period from the second instar larva to middle pupa stages.

To estimate the levels of *white* expression, we visually determined the degree of pigmentation in the eyes (*white*) of 1- to 3-day-old males developing at 25°C, with reference to standard color scales. All flies were scored independently by two observers. On the nine-grade scale for *white*, red (R) eyes corresponded to the wild type and white (W) eyes to the total loss of *white* expression; intermediate pigmentation levels, in order of decreasing gene expression, were brownish red (BrR), brown (Br), dark orange (dOr), orange (Or), dark yellow (dY), yellow (Y) and pale yellow (pY).

### Plasmid construction

The constructs were based on the CaSpeR vector [78]. The pCaSpew15(+RI) plasmid was constructed by inserting an additional *Eco*RI site at +3190 of the *mini-white* gene (*white*) in the pCaSpew15 plasmid. The Wari insulator located on the 3′ side of the *white* gene was deleted from pCaSpew15(+RI) to produce plasmid pCaSpeRΔ700 (CΔ). The 777-bp *white* regulatory sequences containing the testis and eye enhancers from −967 to −1745 relative to the transcription start site (E) were cloned between two frt sites (frt(E)). Insulator fragments (I) were obtained by PCR amplification. The 340-bp fragment containing the Su(Hw)-binding region (Gy) was PCR amplified from the *gypsy* retrotransposon. The 0.858-kb Fab-7 fragment (F7) was cloned by PCR amplification between primers 5′-GATTTCAAGCTGTGTGGCGGGG-3′ and 5′-CGTGAGCGACCGAAACTC-3′. The products after sequencing were subcloned in pSK plasmid, or between lox (lox(I)) or frt sites (frt(I)).

(E)(I)W: The frt(E) fragment was inserted in front of the *white* promoter into the CΔ plasmid digested with *Xba*I ((E)W). The lox(F7) or lox(Gy) fragment was cloned in the middle of the 480-bp *Pvu*I–*Pvu*I fragment from *lacZ* cDNA digested with *Eco*RV. The resulting DNA fragment was inserted into the (E)W digested with *Bam*HI. In the plasmid, the insulators were at −695 relative to the *white* transcription start site.

(I)(E)I^R^W: The lox(F7) or lox(Gy) fragment was cloned into the (E)W plasmid digested with *Pst*I just upstream of the eye enhancer ((I)(E)W). The second insulator fragment was cloned in the middle of 480-bp *lacZ* spacer (I_sp_) and then was inserted into the (I)(E)W plasmid between the eye enhancer and promoter at −345 relative to the *white* transcription start site. As a result, the proximal insulator was located at 585 bp from the transcription start site.

E(Gy)W(Gy) and E(Gy)^R^W(Gy): The eye enhancer was cloned into the CΔ plasmid digested with *Xba*I (EW). The lox(Gy) fragment was cloned in two orientations in the EW plasmid between the eye enhancer and the promoter at −482 relative to the *white* transcription start site. The frt(Gy) fragment was cloned into the EW plasmid digested with *Eco*RI in direct orientation relative to the *white* gene.

E_4,6_(Gy)^R^W(Gy): The frt(Gy) fragment was cloned in EW digested with *Eco*RI (EW(Gy)). The 4.6-kb *Bam*HI-*Bgl*II fragment of the *yellow* gene was cloned in the EW(Gy) plasmid digested with *Bam*HI between the eye enhancer and the promoter (E_4,6_W(Gy)). The lox(Gy) fragment was cloned in reverse orientation in the E_4,6_W plasmid digested with *Bgl*II between the eye enhancer and the promoter at −482 relative to the *white* transcription start site.

E(Gy)^R^_4,6_W(Gy): This construct was made like E_4, 6_(Gy)^R^W(Gy), but the lox(Gy) fragment was cloned in reverse orientation in E_4, 6_W(Gy) restricted with *Bam*HI between the *white* enhancer and promoter in position −5096 relative to the *white* transcription start site.

(Gy)E_4,6_(Gy)W and (Gy)^R^E_ 4,6_(Gy)W: The 4.6-kb *Bam*HI–*Bgl*II fragment of the *yellow* coding region was cloned in EW digested with *Bam*HI upstream of the *white* promoter (E_4,6_W). The lox(Gy) fragment was cloned in both orientations into the E_4,6_W plasmid digested with *Pst*I upstream of the eye enhancer. The frt(Gy) fragment was cloned in direct orientation in position −462 relative to the *white* transcription start site (considering one frt site).

(Gy)_2,1_(E)_2,2_GyW and (Gy)^R^_2,1_(E)_2,2_GyW: The 4.6-kb *Bam*HI–*Bgl*II fragment of the *yellow* coding region was cloned in CΔ digested with *Bam*HI in front of the *white* promoter (_4,6_W). The *lacZ* gene was cloned in pBluSK; frt(E) was cloned approximately in the center of the *lacZ* gene digested with *Eco*47III (_1,4_frt(E)_2,1_pSK). The *Kpn*I–*Bam*HI fragment was then cloned in the _4,6_W plasmid digested with *Kpn*I–*Bgl*II (_1,8_frt(E)_2,4_W). The Gy_sp_ fragment was inserted in the _1,4_frt(E)_2,4_W plasmid digested with *Sma*I in front of the *white* promoter (_1,8_frt(E)_2,2_GyW). The lox(Gy) fragment was inserted into the _1,8_frt(E) _2,2_GyW plasmid digested with *Xba*I in both orientations.

(Gy)_2,1_(E)_2,2_Gy_2,0_W: The construct was made like (Gy)_2,1_(E)_2,2_GyW, but the 1460-bp *Eco*RI–*Bgl*II *yellow* fragment was inserted between proximal Gy and *white* promoter. As a result, the distance between the proximal *gypsy* insulator and *white* transcription start site was 2055 bp.

### Chromatin immunoprecipitation

Chromatin was prepared from mid-late pupae. A 500-mg sample was ground in a mortar in liquid nitrogen and resuspended in 10 mL of buffer A (15 mM HEPES-KOH, pH 7.6; 60 mM KCl, 15 mM NaCl, 13 mM EDTA, 0.1 mM EGTA, 0.15 mM spermine, 0.5 mM spermidine, 0.5% NP-40, 0.5 mM DTT) supplemented with 0.5 mM PMSF and Complete (EDTA-free) Protease Inhibitor Cocktail V (Calbiochem, United States). The suspension was then homogenized in a Dounce homogenizer with pestle B and filtered through Nylon Cell Strainer (BD Biosciences, United States). The homogenate was transferred to 3 mL of buffer A with 10% sucrose (AS), and the nuclei were pelleted by centrifugation at 4 000 *g*, 4°C for 5 min. The pellet was resuspended in 5 mL of buffer A, homogenized again in a Dounce homogenizer, and transferred to 1.5 mL of buffer AS to collect the nuclei by centrifugation. The nuclear pellet was resuspended in wash buffer (15 mM HEPES-KOH, pH 7.6; 60 mM KCl, 15 mM NaCl, 1 mM EDTA, 0.1 mM EGTA, 0.1% NP-40, protease inhibitors) and cross-linked with 1% formaldehyde for 15 min at room temperature. Cross-linking was stopped by adding glycine to a final concentration of 125 mM. The nuclei were washed with three 10-mL portions of wash buffer and resuspended in 1.5 mL of nuclei lysis buffer (15 mM HEPES, pH 7.6; 140 mM NaCl, 1 mM EDTA, 0.1 mM EGTA, 1% Triton X-100, 0.5 mM DTT, 0.1% sodium deoxycholate, 0.1% SDS, protease inhibitors). The suspension was sonicated on ice with a Branson Sonifier 150 (Branson Instruments, United States) for 5×20 sec at 1-min intervals. Debris was removed by centrifugation at 14000 *g*, 4°C for 10 min, and chromatin was pre-cleared in Protein G agarose (Pierce, Unites States) blocked with BSA and salmon sperm DNA. Aliquots of such pre-cleared chromatin were used as the input samples. These samples were incubated overnight, at 4°C, with rat antibodies against Zeste (1∶100), Su(Hw) (1∶500), and CP190 (1∶500); rabbit antibodies against Mod(mdg4)-67.2 (1∶500) and Ey2 (1∶200); and nonspecific IgG purified from rat and rabbit preimmune sera. Chromatin–antibody complexes were collected using blocked protein G (for rat probes) or A (for rabbit probes) agarose at 4°C over 5 h. After several rounds of washing with lysis buffer (as such and with 500 mM NaCl), LiCl buffer (20 mM Tris-HCl, pH 8; 250 mM LiCl, 1 mM EDTA, 0.5% NP-40, 0.5% sodium deoxycholate, protease inhibitors), and TE buffer, the DNA was eluted with elution buffer (50 mM Tris-HCl, pH 8; 1 mM EDTA, 1% SDS), the cross-links were reversed, and the precipitated DNA was extracted by the phenol–chloroform method. The enrichment of specific DNA fragments was analyzed by real-time PCR, using a StepOne Plus Thermal Cycler (Applied Biosystems, United States). Relative enrichment was calculated as a percentage of the input normalized to a control positive genomic site (region 62D for Su(Hw), Cp190, Mod(mdg4)-67.2, and EY2 [36], and PRE from the *Ubx* gene for Zeste [79]. The primers used for PCR in ChIP experiments for genome fragments are shown in Table S1.

### Chromosome conformation capture assay

The 3C assay was performed as described [54], with minimal modifications. The nuclear pellet prepared from 50-mg sample of pupae (see *Chromatin Immunoprecipitation*) was washed with wash buffer and resuspended in 5 mL of nucleus preparation (NP) buffer (15 mM HEPES, pH 7.6; 60 mM KCl, 15 mM NaCl, 4 mM MgCl_2_, 0.1% Triton X-100, 0.5 mM DTT, 1× Complete (EDTA-free) Protease Inhibitor Cocktail V (Calbiochem, United States), and 2% formaldehyde). The suspension was incubated with slow agitation for a total cross-linking time of 30 min at 25°C. Fixation was stopped by adding 2.5 M glycine to a concentration of 0.125 M, and the sample was cooled on ice for at least 5 min and centrifuged at 2500 *g*, 4°C for 5 min. The pellet was washed with two portions of cold NP buffer and one portion of cold 1.25× NEBuffer 3 (62.5 mM Tris-HCl, pH 8.0; 125 mM NaCl, 12.5 mM MgCl_2_, 1.25 mM DTT) (New England Biolabs, United States) and resuspended in 300 µL of 1.25× NEBuffer 3. The suspension was supplemented with 4.5 µL of 20% SDS and incubated at the thermoshaker at 37°C and 1400 rpm for 1 h; then 34 µL of 20% Triton X-100 was added, and the sample was incubated again at 37°C and 1400 rpm for 1 h. At this stage, a 30-µL aliquot of the sample was taken to be used as the undigested control. Thereafter, 1500 units of DpnII (New England Biolabs) was added, and the sample was incubated at 37°C and 1400 rpm for 10 h and then at 65°C and 1400 rpm for 20 min to inactivate DpnII. At this stage, a 30-µL aliquot of the sample was taken to be used as the digested control. Another 170-µL aliquot was cooled, mixed with 250 µL of 1.7× ligation buffer (1.7× T4 DNA ligase buffer with 100 units/mL of T4 DNA Ligase, New England Biolabs), and incubated with slow agitation at 25°C for 5 h. At this stage, a 75-µL aliquot of the sample was taken to be used as the ligation control. Cross-links were reversed overnight at 65°C and 1400 rpm. The sample was incubated with 4 µL of 10 mg/mL RNase A at 37°C and 1400 rpm for 1 h and then with 11 µL of 20 mg/mL Proteinase K at 56°C and 1400 rpm for 4 h. The sample was extracted with 10 mM Tris-HCl (pH 8.0) saturated phenol, 10 mM Tris-HCl (pH 8.0) saturated phenol/chloroform/isoamyl alcohol, and chloroform/isoamyl alcohol. At the each step, the mixture was centrifuged at 10 000 *g* and room temperature for 10 min. The extracted solution was mixed with 0.1 volume of 3 M AcNa (pH 5.2) containing 35 µg glycogen and 2 volumes of 96% ethanol and incubated overnight at −80°C. DNA was precipitated by centrifugation at 20 000 *g* for 90 min at 4°C. The DNA pellet was washed with 70% ethanol, air dried, and resuspended in 100 µL of 10 mM Tris-HCl (pH 7.5). Sample DNA concentrations were adjusted to 10 ng/µL with 10 mM Tris-HCl (pH 8.0). All control procedures and quantitative analyses were performed as described [80]. As a control template containing all ligation products in equal amounts, we used a BAC clone (BACR06H06, RPCI-98, Roswell Park Cancer Institute Drosophila melanogaster BAC library) covering the site of transgenic construct integration into the genome, which was mixed in equimolar amounts with plasmid DNA construct digested with DpnII (New England Biolabs) at a concentration of 10 U/µg DNA. Digested DNA was purified by phenol/chloroform extraction and ethanol precipitation as described above, ligated with T4 DNA ligase at 25°C for 5 h, and purified again in the same way.

Primers were designed so as to flank DpnII restriction sites in the transgenic construct. TaqMan Probes were designed with 5′FAM reporter dye and 3′BHG1 quencher dye. To normalize for the PCR efficiency of different primer pair/probe combinations, standard curves for each combination were generated using the BAC control template. Interaction frequencies were calculated on the basis of Ct values of each sample relative to the standard curve for the given primer pair/probe combination. The primers and probes used in the study are listed in Table S1.

All real-time PCR reactions were carried out in a StepOne Plus system (Applied Biosystems) according to the manufacturer's instructions, in four replications each. Amplification involved initial denaturation at 95°C for 15 min followed by 50 cycles of 95°C for 15 s and 60°C for 60 s. To compare interaction frequencies, the normalization procedure was performed: the amount of test ligation product was divided by the amount of reference product to give a “relative interaction frequency.” Loading adjustment was performed as described [81] to provide for normalization and subsequent comparison of probes from different transgenic flies.

### Western blotting

A sample of 20 flies was ground with a homogenizer in extraction buffer (20 mM HEPES, pH 7.5; 100 mM KCl, 5% glycerol, 10 mM EDTA, 0.1% Triton X-100, 1 mM DTT, 0.5 mM PMSF, 20 mg/mL aprotinin, 5 mg/mL leupeptin, 5 mg/mL pepstatin A), 10 µL per fly). Debris was removed by centrifugation at 12 000 g, 4°C for 10 min, and the appropriate amount of 5× SDS loading was added directly to the homogenate. The sample was boiled for 5 min, centrifuged at 12 000 *g* for 5 min, and loaded onto SDS-PAGE.

### Antibodies

Mouse anti-lamin antibody ADL67.10 (working dilution for Western blotting, 1∶1000) was from the Hybridoma Bank at the University of Iowa. Rabbit anti-Mod(mdg4)-67.2 antibody (1∶5000) was a gift from A. Golovnin. Rat antibodies against α-Su(Hw) (1∶1000), α-CP190 (1∶1000), α-Zeste (1∶200), and E(y)2 (1∶200) were raised in our laboratory and affinity purified. To this end, protein fragments 6× His-Su(Hw) (aa 1–150), CP190 (aa 308–1096), Zeste (aa 1–175) and EY2 were expressed in BL21 cells, affinity purified on Ni-NTA agarose (Invitrogen) according to the manufacturer's protocol, and injected into rats following the standard immunization procedure.

### RNA isolation and real-time PCR

Total RNA was isolated using the TRI reagent (Molecular Research Center, United States) according to the manufacturer's instructions. RNA was treated with two units of Turbo DNase I (Ambion) for 30 min at 37°C to eliminate genomic DNA. The synthesis of cDNA was performed using 2 µg of RNA, ArrayScript reverse transcriptase (Ambion) and oligo(dT) as a primer. The amounts of specific cDNA fragments were quantified by real-time PCR. At least three independent experiments with each primer set were performed for three independent RNA samples. Relative levels of mRNA expression were calculated in the linear amplification range by calibration to a standard curve of genomic DNA to account for differences in primer efficiencies. Individual expression values were normalized with reference to rpl32 mRNA.

### Preparation of the nuclear extract and immunoprecipitation

The nuclear extracts were obtained from S2 cells, and the protein complexes were immunoprecipitated from the extracts. For this purpose, 1×10^8^ S2 cells were washed twice in 10 mL of ice cold PBS, resuspended in 10 mL of ice cold IP-Sucrose buffer (10 mM Tris, pH 7.5; 10 mM NaCl, 10 mM MgCl_2_, 1 mM EDTA, 1 mM EGTA, 1 mM DTT, 250 mM sucrose, 0.5 mM PMSF) with Complete (EDTA-free) Protease Inhibitor Cocktail V (Calbiochem, United States), incubated on ice for 10 min, and homogenized with a Dounce loose pestle (20 strokes). The nuclei were then pelleted by centrifugation at 3000 *g*, 4°C for 10 min. The pellet was resuspended in 500 µL of ice cold IP-10 buffer (10 mM Tris, pH 7.5; 10 mM NaCl, 10 mM MgCl_2_, 1 mM EDTA, 1 mM EGTA, 1 mM DTT;,0.1% NP-40, 10% glycerol, 0.5 mM PMSF, and Complete Protease Inhibitor Cocktail V), homogenized with a Dounce tight pestle (20 strokes), and mixed with an equal volume of IP-850 buffer (10 mM Tris, pH 7.5; 850 mM NaCl, 10 mM MgCl_2_, 1 mM EDTA, 1 mM EGTA, 1 mM DTT, 0.1% NP-40, 10% glycerol, 0.5 mM PMSF, and Complete Protease Inhibitor Cocktail V). The suspension was incubated on ice for 10 minutes and then centrifuged at 20 000 rpm, 4°C, for 10 min. The supernatant fluid (the nuclear fraction) was collected for immunoprecipitation experiments.

Rat antibodies against α-Su(Hw) (1∶200) and α-Zeste (1∶100) were conjugated with Protein G agarose, and rabbit antibodies against α-Mod(mdg4)-67.2 (1∶500), with Protein A agarose beads (Pierce); in respective control experiments, rat or rabbit preimmune serum was used. An aliquot of an antibody was mixed with 30 µL of agarose beads equilibrated in IP buffer with 150 mM NaCl (IP-150) and incubated on a rotator at 4°C for 3 h. The beads were then washed with IP-150, blocked with 1% BSA for 30 min under the same conditions, and washed with two portions of IP-150. The nuclear extract was adjusted to 150 mM NaCl, and its 1 ml containing approximately 1 mg of total protein was mixed with 30 µL of “fresh” agarose beads equilibrated in IP-150 and incubated at 4°C for 1 h for pre-clearing the sample. The beads were pelleted, and the supernatant fluid was transferred to a new tube and mixed with antibody-conjugated beads. The samples were incubated on a rotary mixer at 4°C for 3 h, and the beads were washed with three portions of IP buffer with 300 mM NaCl, one portion of IP buffer with 500 mM NaCl, and one portion of IP buffer with 150 mM NaCl. After the last washing step, the beads were resuspended in SDS–PAGE loading buffer, boiled, and analyzed by Western blotting. Proteins were detected using the SuperSignal West Fempto substrate (Pierce).

### Yeast two-hybrid assay

Yeast two-hybrid assay was carried out using yeast strain pJ69-4A, with plasmids and protocols from Clontech. For growth assays, plasmids were transformed into yeast strain pJ69-4A by the lithium acetate method, as described by the manufacturer, and plated on media without tryptophan and leucine. After 2 days of growth at 30°C, the cells were plated on selective media without tryptophan, leucine, histidine and adenine, and their growth was compared after 2–3 days. Liquid culture β-galactosidase assay was performed according to the yeast protocols handbook (Clontech). Each assay was repeated twice.

## Supporting Information

Figure S1 Testing role of Zeste in activity of eye enhancer. Role of the *z^v77h^* mutation in expression of *white* in transgenic lines and their derivatives carrying (A) *lox*-flanked *gypsy* insulator and the *frt*-flanked eye enhancer or (B) lox-flanked Fab-7 insulator and the frt-flanked eye enhancer. In the reductive scheme of the transgenic construct used in the assay, the *white* gene is shown as white box with an arrow indicating the direction of transcription; the triangle indicates deletion of the Wari insulator located at the 3′ end of the *white* gene; downward arrows indicate target sites for Flp recombinase (*frt*) or Cre recombinase (*lox*); the same sites in construct names are denoted by parentheses; the eye enhancer (E) is shown as white rectangle; the direction of the *gypsy* insulator (Gy) is indicated by the apex of the pentagon; the F7 insulator (F7) is indicated by black rectangle. The numbers of transgenic lines with different levels of *white* pigmentation in the eyes are indicated. Arrows indicate the excision of an element to produce the derivative transgenic lines. Wild-type *white* expression determined the bright red eye color (R); in the absence of *white* expression, the eyes were white (W). Intermediate levels of pigmentation, with the eye color ranging from pale yellow (pY), through yellow (Y), dark yellow (dY), orange (Or), dark orange (dOr), and brown (Br) to brownish red (BrR), reflect the increasing levels of *white* expression. N is the number of lines in which flies acquired a new eye color phenotype by deletion (Δ) of the specified DNA fragment; T is the total number of lines examined for each particular construct. *z^v77h^*, a null-mutation of the *zeste* gene.
(TIF)

Figure S2 Phenotypic effects of the *mod(mdg4)^u1^* mutation and overexpression of the Su(Hw) protein. (A) Western analysis of the *y^1^w^1118^* line and three transgenic lines carrying *hsp70-su(Hw)* transgene, numbered 1, 2, and 3. Extracts from individual middle pupae were loaded onto each lane and probed with C-terminal specific anti-Su(Hw) antibodies. Anti-dCTCF antibody was used as control. Heat shock (hs) treatment of pupae was performed for 2 hours. (B) Structural scheme of the *ct^6^* allele: the bent arrow indicates the start site and direction of *cut* gene transcription, the gray rectangle is the wing margin enhancer (En-wm), and the triangle shows the insertion of *gypsy* with flanking LTRs (small black rectangles) and the insulator (hatched rectangle). Effects of the *mod(mdg4)^u1^* mutation and the combination of *mod(mdg4)^u1^* with *hsp70su(Hw)* on the cut wing phenotype in flies with the *ct^6^* allele are shown.
(TIF)

Figure S3 The role of Su(Hw) and Mod(mdg4)-67.2 in the eye enhancer blocking by the *gypsy* insulator. To induce Su(Hw) overexpression (+ *hsp70su(Hw)*), transgenic flies carrying the *hsp70su(Hw)* construct were treated by heat shock as described in Material and Methods. Designation “mod/mod” refers to transgenic lines homozygous for the *mod(mdg4)^u1^* or *mod(mdg4)^T6^* mutation. An asterisk indicates variegated eye pigmentation. Other designations are as in Figure S1 and S2.
(TIF)

Figure S4 Role of the *gypsy* insulator located on the 3′ side of the *white* gene and Mod(mdg4)-67.2 in stimulation of *white* expression. The derivatives carrying one copy of the *gypsy* insulator from the 3′ side of the *white* gene were tested. The original and derivatives transgenic lines are described in Figure 6. In (A) the enhancer is located near the *white* gene. In (B) and (C) the enhancer is located at the 4.6 kb distance from the promoter. Designations are as in Figure S1 and S3.
(TIF)

Figure S5 The results of ChIP of specified chromatin regions with antibodies to Zeste in two transgenic lines carrying constructs with two copies of the *gypsy* insulators. Designations: Ee (the eye enhancer), Pw (promoter), W (coding region of the *white* gene) and Gd (distal *gypsy* insulator). The *rpl32* and *tubulin* (tub) coding regions were used as controls devoid of Zeste binding sites. Other designations are as in Figure S1.
(TIF)

Figure S6 Testing for the direct influence of the *mod(mdg4)^u1^* mutation on the expression of Zeste. (A) Relative levels of the *zeste* gene expression in wild-type (WT) and *mod(mdg4)^u1^* backgrounds. The transcripts were isolated from 2-day pupae and quantified by RT-PCR, with *tubulin* (*tub*) expression being used as a control. The transcript levels were normalized relative to that of *rpl32*. Error bars standard deviations of triplicate measurements. (B) Levels of the Zeste protein in wild-type (WT) and *mod(mdg4)^u1^* pupae tested by Western blot analysis. Lamin was used as a loading control.
(TIF)

Figure S7 The effect of Mod(mdg4)-67.2 on the level of Zeste in transgenic lines. (A) The results of ChIP (percentages of input DNA normalized relative to the endogenous positive binding site for Zeste from the *Ubx* promoter region) of specified chromatin regions with antibodies to Zeste in derivatives of two transgenic lines carrying the eye enhancer in close proximity to the *white* promoter in the wild-type and *mod(mdg4)^u1^* (*m/m*) mutant backgrounds. Phenotypes of these derivative lines are described in Figure S4A. (B) The results of ChIP with antibodies to Zeste in derivatives of the transgenic line described in Figure 8 in the wild-type and the *mod(mdg4)^u1^* backgrounds. Phenotypes corresponding for these lines are described in Figure S4B. (C) The results of ChIP with antibodies to Zeste in derivatives of two transgenic line carrying the eye enhancer at 4.6 kb from the *white* promoter in the wild-type and the *mod(mdg4)^u1^* backgrounds. Phenotypes corresponding to these lines are described in Figure S4C. Other designations are as in Figures S4 and S5.
(TIF)

Table S1 Oligos used in the study. Table summarizes information about oligos used in ChIP, 3C and RT-PCR experiments.
(DOC)

## References

[1] Kuhn E J, Geyer PK (2003) Genomic insulators: Connecting properties to mechanism. Curr Opin Cell Biol 15: 259–265. 12787766 10.1016/s0955-0674(03)00039-5

[2] Brasset E, Vaury C (2005) Insulators are fundamental components of the eukaryotic genomes. Heredity 94 6: 571–576. 15815711 10.1038/sj.hdy.6800669

[3] Zhao H, Dean A (2005) Organizing the genome: Enhancers and insulators. Biochem Cell Biol 83: 516–524. 16094455 10.1139/o05-054

[4] Wallace JA, Felsenfeld G (2007) We gather together: Insulators and genome organization. Curr Opin Genet Dev 17: 400–407. 17913488 10.1016/j.gde.2007.08.005 PMC2215060

[5] Valenzuela L, Kamakaka RT (2006) Chromatin insulators. Annu Rev Genet 40: 107–138. 16953792 10.1146/annurev.genet.39.073003.113546

[6] Maksimenko OG, Chetverina DA, Georgiev PG (2006) Insulators of higher eukaryotes: Properties, mechanisms of action, and role in transcription regulation. Russ J Genet 42: 845–857. 17025153

[7] Barkess G, West AG (2012) Chromatin insulator elements: Establishing barriers to set heterochromatin boundaries. Epigenomics 4: 67–80. 22332659 10.2217/epi.11.112

[8] Kyrchanova O, Chetverina D, Maksimenko O, Kullyev A, Georgiev P (2008) Orientation-dependent interaction between *Drosophila* insulators is a property of this class of regulatory elements. Nucleic Acids Res 36: 7019–7028. 18987002 10.1093/nar/gkn781 PMC2602758

[9] Maksimenko O, Golovnin A, Georgiev P (2008) Enhancer-promoter communication is regulated by insulator pairing in a *Drosophila* model bigenic locus. Mol Cell Biol 28: 5469–5477. 18573869 10.1128/MCB.00461-08 PMC2519739

[10] Chetverina D, Savitskaya E, Maksimenko O, Melnikova L, Zaytseva O (2008) Red flag on the *white* reporter: A versatile insulator abuts the *white* gene in *Drosophila* and is omnipresent in *mini-white* constructs. Nucleic Acids Res 36: 929–37. 18086699 10.1093/nar/gkm992 PMC2241909

[11] Kravchenko E, Savitskaya E, Kravchuk O, Parshikov A, Georgiev P, et al (2005) Pairing between *gypsy* insulators facilitates the enhancer action in *trans* throughout the *Drosophila* genome. Mol Cell Biol 25: 9283–9291. 16227580 10.1128/MCB.25.21.9283-9291.2005 PMC1265844

[12] Li HB, Muller M, Bahechar IA, Kyrchanova O, Ohno K, et al (2011) Insulators, not Polycomb Response Elements, are required for long-distance interactions between Polycomb targets in *Drosophila melanogaster* . Mol Cell Biol 31: 616–625. 21135119 10.1128/MCB.00849-10 PMC3028641

[13] Adryan B, Woerfel G, Birch-Machin I, Gao S, Quick M, et al (2007) Genomic mapping of Suppressor of Hairy-wing binding sites in *Drosophila* . Genome Biol 8: R167. 17705839 10.1186/gb-2007-8-8-r167 PMC2374998

[14] Nègre N, Brown CD, Shah PK, Kheradpour P, Morrison CA, et al (2010) A comprehensive map of insulator elements for the *Drosophila* genome. PloS Genet 6: e1000814. 20084099 10.1371/journal.pgen.1000814 PMC2797089

[15] Nègre N, Brown CD, Ma L, Bristow CA, Miller S, et al (2011) A *cis*-regulatory map of the *Drosophila* genome. Nature 471: 527–531. 21430782 10.1038/nature09990 PMC3179250

[16] Bartkuhn M, Staub T, Herold M, Herrmann M, Rathke C, et al (2009) Active promoters and insulators are marked by the centrosomal protein 190. EMBO J 28: 877–888. 19229299 10.1038/emboj.2009.34 PMC2670862

[17] Roy S, Ernst J, Kharchenko PV, Kheradpour P, Negre N, et al (2010) Identification of functional elements and regulatory circuits by *Drosophila* modENCODE. Science 330: 1787–1797. 21177974 10.1126/science.1198374 PMC3192495

[18] Kehayova P, Monahan K, Chen W, Maniatis T (2011) Regulatory elements required for the activation and repression of the protocadherin-{alpha} gene cluster. Proc Natl Acad Sci USA 108: 17195–17200. 21949399 10.1073/pnas.1114357108 PMC3193253

[19] Liu Z, Scannell DR, Eisen MB, Tjian R (2011) Control of embryonic stem cell lineage commitment by core promoter factor, TAF3. Cell 146: 720–731. 21884934 10.1016/j.cell.2011.08.005 PMC3191068

[20] Handoko L, Xu H, Li G, Ngan CY, Chew E, et al (2011) CTCF-mediated functional chromatin interactome in pluripotent cells. Nature Genet 43: 630–638. 21685913 10.1038/ng.857 PMC3436933

[21] Dixon JR, Selvaraj S, Yue F, Kim A, Li Y, et al (2012) Topological domains in mammalian genomes identified by analysis of chromatin interactions. Nature 485: 376–380. 22495300 10.1038/nature11082 PMC3356448

[22] Sexton T, Yaffe E, Kenigsberg E, Bantignies F, Leblanc B, et al (2012) Three-dimensional folding and functional organization principles of the *Drosophila* genome. Cell 148: 458–472. 22265598 10.1016/j.cell.2012.01.010

[23] Kellum R, Schedl P (1991) A position-effect assay for boundaries of higher order chromosomal domains. Cell 64: 941–950. 1848159 10.1016/0092-8674(91)90318-s

[24] Kellum R, Schedl P (1992) A group of scs elements function as domain boundaries in an enhancer-blocking assay. Mol Cell Biol 12: 2424–2431. 1569958 10.1128/mcb.12.5.2424-2431.1992 PMC364415

[25] Hagstrom K, Muller M, Schedl P (1996) Fab-7 functions as a chromatin domain boundary to ensure proper segment specification by the *Drosophila bithorax* complex. Genes Dev 10: 3202–3215. 8985188 10.1101/gad.10.24.3202

[26] Zhou J, Barolo S, Szymanski P, Levine M (1996) The Fab-7 element of the bithorax complex attenuates enhancer-promoter interactions in the *Drosophila* embryo. Genes Dev 10: 3195–3201. 8985187 10.1101/gad.10.24.3195

[27] Barges S, Mihaly J, Galloni M, Hagstrom K, Muller M, et al (2000) The *Fab-8* boundary defines the distal limit of the *bithorax* complex *iab*-7 domain and insulates *iab*-7 from initiation elements and a PRE in the adjacent *iab*-8 domain. Development 127: 779–790. 10648236 10.1242/dev.127.4.779

[28] Schweinsberg S, Schedl P (2004) Developmental modulation of Fab-7 boundary function. Development 131: 4743–4749. 15329342 10.1242/dev.01343

[29] Gruzdeva N, Kyrchanova O, Parshikov A, Kullyev A, Georgiev P (2005) The Mcp element from the *bithorax* complex contains an insulator that is capable of pairwise interactions and can facilitate enhancer–promoter communication. Mol Cell Biol 25: 3682–3689. 15831473 10.1128/MCB.25.9.3682-3689.2005 PMC1084309

[30] Belozerov VE, Majumder P, Shen P, Cai HN (2003) A novel boundary element may facilitate independent gene regulation in the *Antennapedia* complex of *Drosophila* . EMBO J 22: 3113–3121. 12805225 10.1093/emboj/cdg297 PMC162149

[31] Conte C, Dastugue B, Vaury C (2002) Coupling of enhancer and insulator properties identified in two retrotransposons modulates their mutagenic impact on nearby genes. Mol Cell Biol 22: 1767–1777. 11865056 10.1128/MCB.22.6.1767-1777.2002 PMC135603

[32] Holdridge C, Dorsett D (1991) Repression of *hsp70* heat shock gene transcription by the suppressor of hairy-wing protein of *Drosophila melanogaster* . Mol Cell Biol 11: 1894–1900. 1900919 10.1128/mcb.11.4.1894-1900.1991 PMC359869

[33] Geyer PK, Corces VG (1992) DNA position-specific repression of transcription by a *Drosophila* zinc finger protein. Genes Dev 6: 1865–1873. 1327958 10.1101/gad.6.10.1865

[34] Golovnin A, Biryukova I, Romanova O, Silicheva M, Parshikov A, et al (2003) An endogenous Su(Hw) insulator separates the *yellow* gene from the *Achaete-scute* gene complex in *Drosophila* . Development 130: 3249–3258. 12783795 10.1242/dev.00543

[35] Parnell TJ, Viering MM, Skjesol A, Helou C, Kuhn EJ, et al (2003) An endogenous suppressor of hairy-wing insulator separates regulatory domains in *Drosophila* . Proc Natl Acad Sci U S A 100: 13436–13441. 14597701 10.1073/pnas.2333111100 PMC263832

[36] Parnell TJ, Kuhn EJ, Gilmore BL, Helou C, Wold MS, et al (2006) Identification of genomic sites that bind the *Drosophila* suppressor of Hairy-wing insulator protein. Mol Cell Biol 26: 5983–5993. 16880510 10.1128/MCB.00698-06 PMC1592791

[37] Herold M, Bartkuhn M, Renkawitz R (2012) CTCF: Insights into insulator function during development. Development 139: 1045–1057. 22354838 10.1242/dev.065268

[38] Cai HN, Shen P (2001) Effects of *cis* arrangement of chromatin insulators on enhancer-blocking activity. Science 291: 493–495. 11161205 10.1126/science.291.5503.493

[39] Muravyova E, Golovnin A, Gracheva E, Parshikov A, Belenkaya T, et al (2001) Loss of insulator activity by paired Su(Hw) chromatin insulators. Science 291: 495–498. 11161206 10.1126/science.291.5503.495

[40] Blanton J, Gaszner M, Schedl P (2003) Protein : protein interactions and the pairing of boundary elements in vivo. Genes Dev 17: 664–675. 12629048 10.1101/gad.1052003 PMC196003

[41] Ameres SL, Drueppel L, Pfleiderer K, Schmidt A, Hillen W, et al (2005) Inducible DNA-loop formation blocks transcriptional activation by an SV40 enhancer. EMBO J 24: 358–367. 15650749 10.1038/sj.emboj.7600531 PMC545818

[42] Bondarenko VA, Liu YV, Jiang YI, Studitsky VM (2003) Communication over a large distance: Enhancers and insulators. Biochem Cell Biol 81: 241–251. 12897858 10.1139/o03-051

[43] Bondarenko VA, Jiang YI, Studitsky VM (2003) Rationally designed insulator-like elements can block enhancer action in vitro. EMBO J 22: 4728–4737. 12970185 10.1093/emboj/cdg468 PMC212734

[44] Mukhopadhyay S, Schedl P, Studitsky VM, Sengupta AM (2011) Theoretical analysis of the role of chromatin interactions in long-range action of enhancers and insulators. Proc Natl Acad Sci U S A 108: 19919–19924. 22123989 10.1073/pnas.1103845108 PMC3250180

[45] Gohl D, Aoki T, Blanton J, Shanower G, Kappes G, et al (2011) Mechanism of chromosomal boundary action: Roadblock, sink, or loop? Genetics 187: 731–748. 21196526 10.1534/genetics.110.123752 PMC3063668

[46] Hou C, Zhao H, Tanimoto K, Dean A (2008) CTCF-dependent enhancer-blocking by alternative chromatin loop formation. Proc Natl Acad Sci U S A 105: 20398–20403. 19074263 10.1073/pnas.0808506106 PMC2629272

[47] Gause M, Morcillo P, Dorsett D (2001) Insulation of enhancer–promoter communication by a *gypsy* transposon insert in the *Drosophila cut* gene: Cooperation between suppressor of hairy-wing and modifier of mdg4 proteins. Mol Cell Biol 21: 4807–4817. 11416154 10.1128/MCB.21.14.4807-4817.2001 PMC87172

[48] Ghosh D, Gerasimova TI, Corces VG (2001) Interactions between the Su(Hw) and Mod(mdg4) proteins required for *gypsy* insulator function. EMBO J 20: 2518–2527. 11350941 10.1093/emboj/20.10.2518 PMC125459

[49] Pai C-Y, Lei EP, Ghosh D, Corces VG (2004) The centrosomal protein CP190 is a component of the *gypsy* chromatin insulator. Mol Cell 16: 737–748. 15574329 10.1016/j.molcel.2004.11.004

[50] Kurshakova M, Maksimenko O, Golovnin A, Pulina M, Georgieva S, et al (2007) Evolutionarily conserved E(y)2/Sus1 protein is essential for the barrier activity of Su(Hw)-dependent insulators in *Drosophila* . Mol Cell 27: 332–338. 17643381 10.1016/j.molcel.2007.05.035

[51] Golovnin A, Mazur A, Kopantseva M, Kurshakova M, Gulak PV, et al (2007) Integrity of the Mod(mdg4)-67.2 BTB domain is critical to insulator function in *Drosophila* . Mol Cell Biol 27: 963–974. 17101769 10.1128/MCB.00795-06 PMC1800699

[52] Bonchuk A, Denisov S, Georgiev P, Maksimenko O (2011) Drosophila BTB/Poz domains of “ttk group” can form multimers and selectively interact with each other. J Mol Biol 412: 423–436. 21821048 10.1016/j.jmb.2011.07.052

[53] Krivega M, Savitskaya E, Krivega I, Karakozova M, Parshikov A, et al (2010) Interaction between a pair of *gypsy* insulators or between *gypsy* and Wari insulators modulates Flp site-specific recombination in *Drosophila melanogaster* . Chromosoma 119: 425–434. 20354861 10.1007/s00412-010-0268-7

[54] Comet I, Schuettengruber B, Sexton T, Cavalli G (2011) A chromatin insulator driving three-dimensional Polycomb response element (PRE) contacts and Polycomb association with the chromatin fiber. Proc Natl Acad Sci U S A 108: 2294–2299. 21262819 10.1073/pnas.1002059108 PMC3038747

[55] Maeda RK, Karch F (2011) Gene expression in time and space: Additive vs. hierarchical organization of *cis*-regulatory regions. Curr Opin Genet Dev 21: 187–193. 21349696 10.1016/j.gde.2011.01.021

[56] Gyurkovics H, Gausz J, Kummer J, Karch F (1990) A new homeotic mutation in the *Drosophila bithorax* complex removes a boundary separating two domains of regulation. EMBO J 9: 2579–2585. 1973385 10.1002/j.1460-2075.1990.tb07439.x PMC552290

[57] Galloni M, Gyurkovics H, Schedl P, Karch F (1993) The bluetail transposon: Evidence for independent *cis*-regulatory domains and domain boundaries in the bithorax complex. EMBO J 12: 1087–1097. 8384551 10.1002/j.1460-2075.1993.tb05750.x PMC413310

[58] Karch F, Galloni M, Sipos L, Gausz J, Gyurkovics H, et al (1994) Mcp and Fab-7: Molecular analysis of putative boundaries of *cis*-regulatory domains in the bithorax complex of *Drosophila melanogaster* . Nucleic Acids Res 22: 3138–3146. 7915032 10.1093/nar/22.15.3138 PMC310287

[59] Rodin S, Kyrchanova O, Pomerantseva E, Parshikov A, Georgiev P (2007) New properties of *Drosophila* Fab-7 insulator. Genetics 177: 113–121. 17890362 10.1534/genetics.107.075887 PMC2013716

[60] Kuhn EJ, Viering MM, Rhodes KM, Geyer PK (2003) A test of insulator interactions in *Drosophila* . EMBO J 22: 2463–2471. 12743040 10.1093/emboj/cdg241 PMC155999

[61] Schweinsberg S, Hagstrom K, Gohl D, Schedl P, Kumar RP, et al (2004) The enhancer-blocking activity of the Fab-7 boundary from the *Drosophila* bithorax complex requires GAGA-factor-binding sites. Genetics 168: 1371–1384. 15579691 10.1534/genetics.104.029561 PMC1448804

[62] Savitskaya E, Melnikova L, Kostuchenko M, Kravchenko E, Pomerantseva E, et al. 2006. Study of long-distance functional interactions between Su(Hw) insulators that can regulate enhancer–promoter communication in *Drosophila melanogaster* . Mol Cell Biol 26: 754–761. 16428433 10.1128/MCB.26.3.754-761.2006 PMC1347022

[63] Melnikova L, Kostuchenko M, Silicheva M, Georgiev P (2008) *Drosophila gypsy* insulator and *yellow* enhancers regulate activity of *yellow* promoter through the same regulatory element. Chromosoma 117: 137–145. 17994318 10.1007/s00412-007-0132-6

[64] Qian S, Varjavand B, Pirrotta V (1992) Molecular analysis of the zeste-*white* interaction reveals a promoter-proximal element essential for distant enhancer-promoter communication. Genetics 131: 79–90. 1375573 10.1093/genetics/131.1.79 PMC1204967

[65] Pirrotta V, Manet E, Hardon E, Bickel SE, Benson M (1987) Structure and sequence of the *Drosophila zeste* gene. EMBO J 6: 791–799. 3582372 10.1002/j.1460-2075.1987.tb04821.x PMC553464

[66] Harrison DA, Gdula DA, Coyne RS, Corces VG (1993) A leucine zipper domain of the suppressor of Hairy-wing protein mediates its repressive effect on enhancer function. Genes Dev 7: 1966–1978. 7916729 10.1101/gad.7.10.1966

[67] Kim J, Shen B, Rosen C, Dorsett D (1996) The DNA-binding and enhancer-blocking domains of the *Drosophila* suppressor of Hairy-wing protein. Mol Cell Biol 16: 3381–3392. 8668153 10.1128/mcb.16.7.3381 PMC231332

[68] Georgiev P, Kozycina M (1996) Interaction between mutations in the suppressor of *Hairy wing* and *modifier of mdg4* genes of *Drosophila melanogaster* affecting the phenotype of *gypsy*-induced mutations. Genetics 142: 425–436. 8852842 10.1093/genetics/142.2.425 PMC1206977

[69] Cai HN, Levine M (1997) The *gypsy* insulator can function as a promoter-specific silencer in the *Drosophila* embryo. EMBO J 16: 1732–1741. 9130717 10.1093/emboj/16.7.1732 PMC1169776

[70] Capelson M, Corces VG (2005) The ubiquitin ligase dTopors directs the nuclear organization of a chromatin insulator. Mol Cell 20: 105–116. 16209949 10.1016/j.molcel.2005.08.031

[71] Kostyuchenko M, Savitskaya E, Koryagina E, Melnikova L, Karakozova M, et al (2009) Zeste can facilitate long-range enhancer–promoter communication and insulator bypass in *Drosophila melanogaster* . Chromosoma 118: 665–674. 19578867 10.1007/s00412-009-0226-4

[72] Raab JR, Kamakaka RT (2010) Insulators and promoters: Closer than we think. Nature Rev Genet 11: 439–446. 20442713 10.1038/nrg2765 PMC3477808

[73] Chopra VS, Cande J, Hong JW, Levine M (2009) Stalled Hox promoters as chromosomal boundaries. Genes Dev 23: 1505–1509. 19515973 10.1101/gad.1807309 PMC2704471

[74] Erokhin M, Davydova A, Kyrchanova O, Parshikov A, Georgiev P, et al (2011) Insulators form gene loops by interacting with promoters in *Drosophila* . Development 138: 4097–4106. 21862564 10.1242/dev.062836

[75] Karess RE, Rubin GM (1984) Analysis of P transposable element functions in *Drosophila* . Cell 38: 135–146. 6088058 10.1016/0092-8674(84)90534-8

[76] Golic KG, Lindquist S (1989) The FLP recombinase of yeast catalyzes site-specific recombination in the *Drosophila* genome. Cell 59: 499–509. 2509077 10.1016/0092-8674(89)90033-0

[77] Siegal ML, Hartl DL (2000) Application of Cre/loxP in *Drosophila*: Site-specific recombination and transgene coplacement. Methods Mol Biol 136: 487–495. 10840736 10.1385/1-59259-065-9:487

[78] Pirrotta V (1988) Vectors for P-mediated transformation in *Drosophila* . Biotechnology 10: 437–456. 2850048 10.1016/b978-0-409-90042-2.50028-3

[79] Benson M, Pirrotta V (1987) The product of the *Drosophila zeste* gene binds to specific DNA sequences in *white* and *Ubx* . EMBO J 6: 1387–1392. 3608982 10.1002/j.1460-2075.1987.tb02379.x PMC553944

[80] Hagège H, Klous P, Braem C, Splinter E, Dekker J, Cathala G, de Laat W, Forné T (2007) Quantitative analysis of chromosome conformation capture assays (3C-qPCR). Nature Protoc 2: 1722–1733. 17641637 10.1038/nprot.2007.243

[81] Moshkovich N, Nisha P, Boyle PJ, Thompson BA, Dale RK, Lei EP (2011) RNAi-independent role for Argonaute2 in CTCF/CP190 chromatin insulator function. Genes Dev 25: 1686–1701. 21852534 10.1101/gad.16651211 PMC3165934

