# Supplementary figures and images for: Effective Blocking of the White Enhancer Requires Cooperation between Two Main Mechanisms Suggested for the Insulator Function
Source: PLoS Genet. 2013 Jul 4;9(7):e1003606. doi: 10.1371/journal.pgen.1003606 (PMC3701704; doi:10.1371/journal.pgen.1003606)

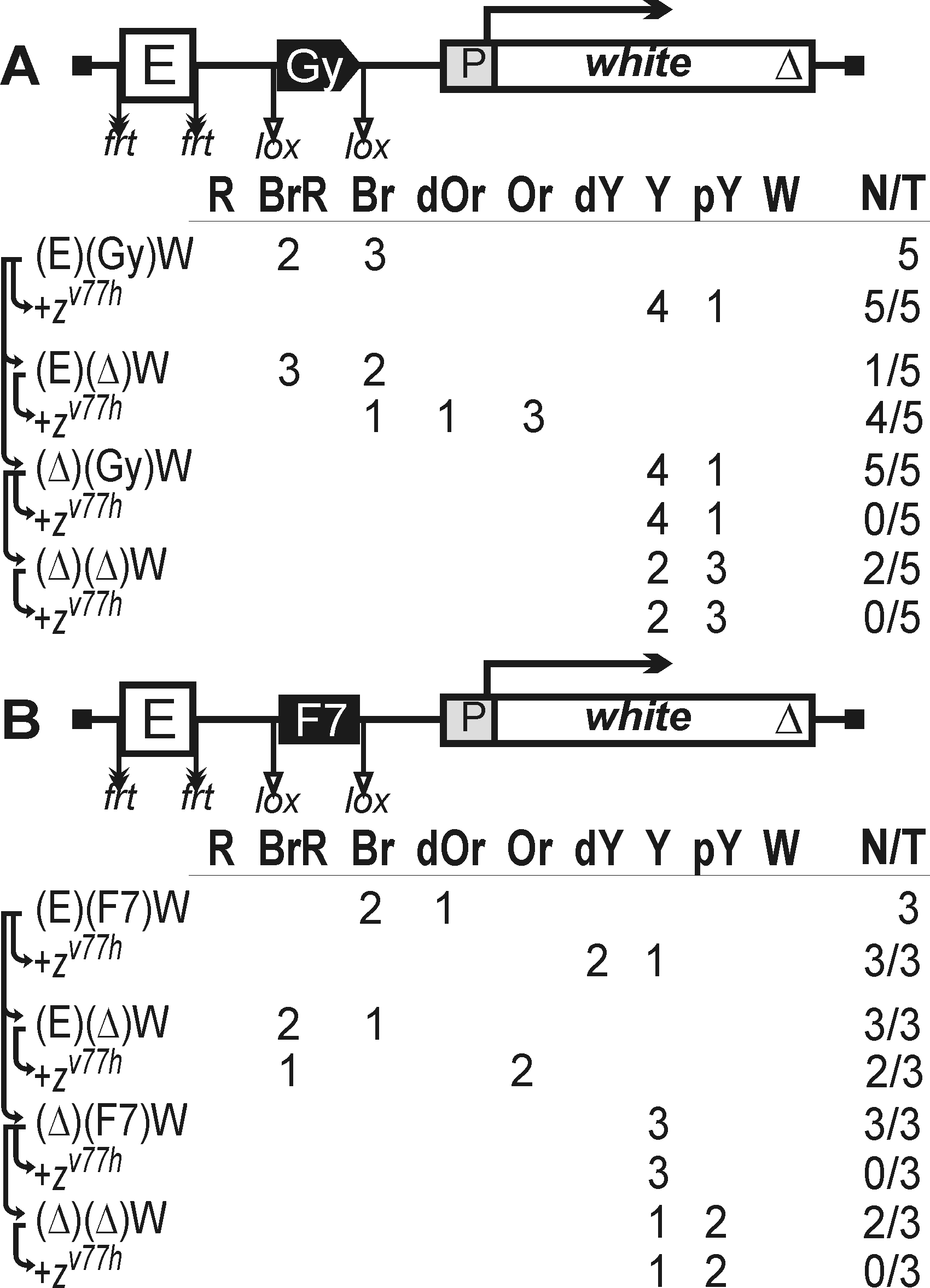

Supplement: Figure S1 — Testing role of Zeste in activity of eye enhancer. Role of the zv77h mutation in expression of white in transgenic lines and their derivatives carrying (A) lox-flanked gypsy insulator and the frt-flanked eye enhancer or (B) lox-flanked Fab-7 insulator and the frt-flanked eye enhancer. In the reductive scheme of the transgenic construct used in the assay, the white gene is shown as white box with an arrow indicating the direction of transcription; the triangle indicates deletion of the Wari insulator located at the 3′ end of the white gene; downward arrows indicate target sites for Flp recombinase (frt) or Cre recombinase (lox); the same sites in construct names are denoted by parentheses; the eye enhancer (E) is shown as white rectangle; the direction of the gypsy insulator (Gy) is indicated by the apex of the pentagon; the F7 insulator (F7) is indicated by black rectangle. The numbers of transgenic lines with different levels of white pigmentation in the eyes are indicated. Arrows indicate the excision of an element to produce the derivative transgenic lines. Wild-type white expression determined the bright red eye color (R); in the absence of white expression, the eyes were white (W). Intermediate levels of pigmentation, with the eye color ranging from pale yellow (pY), through yellow (Y), dark yellow (dY), orange (Or), dark orange (dOr), and brown (Br) to brownish red (BrR), reflect the increasing levels of white expression. N is the number of lines in which flies acquired a new eye color phenotype by deletion (Δ) of the specified DNA fragment; T is the total number of lines examined for each particular construct. zv77h, a null-mutation of the zeste gene. (TIF) [file pgen.1003606.s001.tif]

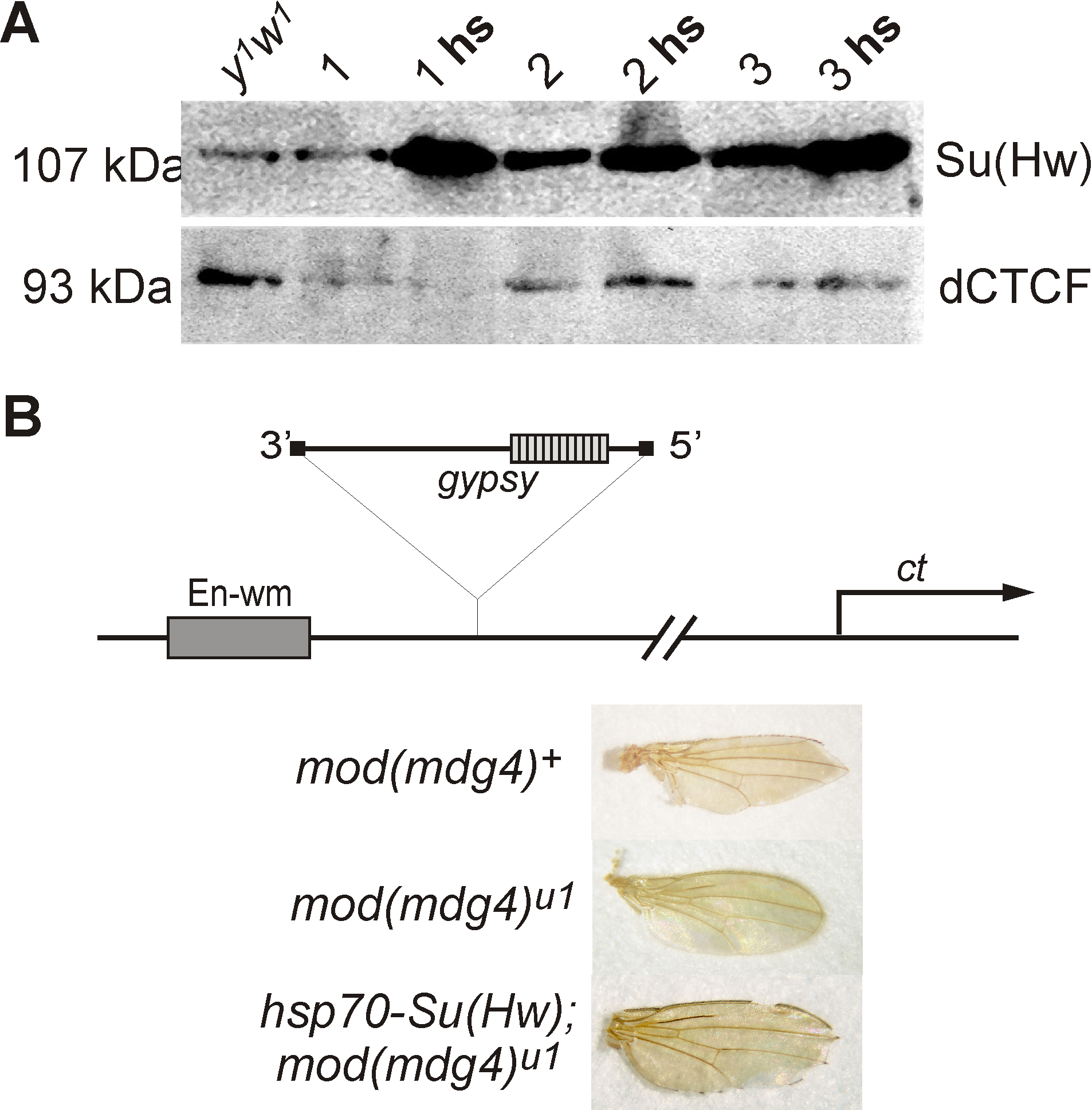

Supplement: Figure S2 — Phenotypic effects of the mod(mdg4)u1 mutation and overexpression of the Su(Hw) protein. (A) Western analysis of the y1w1118 line and three transgenic lines carrying hsp70-su(Hw) transgene, numbered 1, 2, and 3. Extracts from individual middle pupae were loaded onto each lane and probed with C-terminal specific anti-Su(Hw) antibodies. Anti-dCTCF antibody was used as control. Heat shock (hs) treatment of pupae was performed for 2 hours. (B) Structural scheme of the ct6 allele: the bent arrow indicates the start site and direction of cut gene transcription, the gray rectangle is the wing margin enhancer (En-wm), and the triangle shows the insertion of gypsy with flanking LTRs (small black rectangles) and the insulator (hatched rectangle). Effects of the mod(mdg4)u1 mutation and the combination of mod(mdg4)u1 with hsp70su(Hw) on the cut wing phenotype in flies with the ct6 allele are shown. (TIF) [file pgen.1003606.s002.tif]

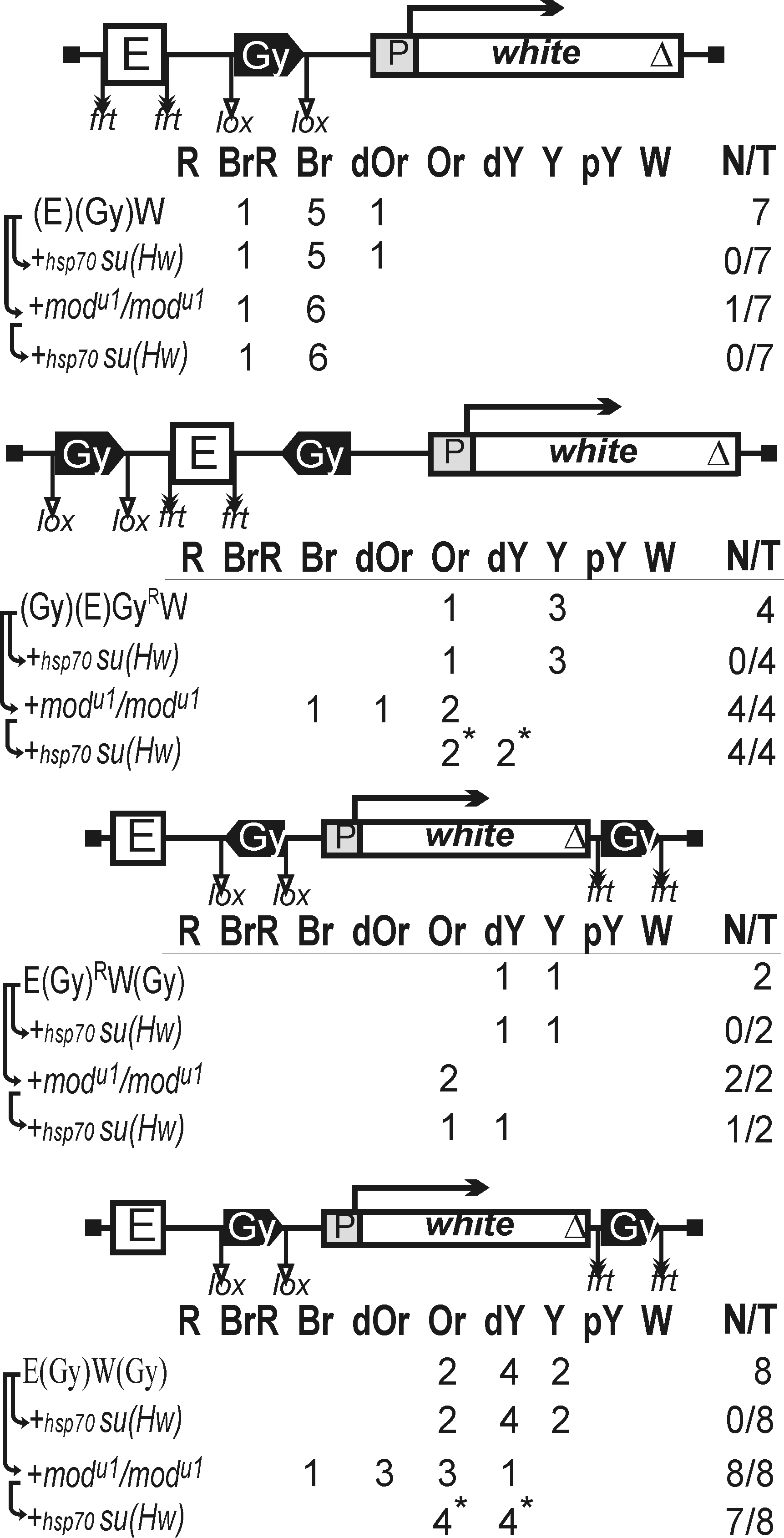

Supplement: Figure S3 — The role of Su(Hw) and Mod(mdg4)-67.2 in the eye enhancer blocking by the gypsy insulator. To induce Su(Hw) overexpression (+ hsp70su(Hw)), transgenic flies carrying the hsp70su(Hw) construct were treated by heat shock as described in Material and Methods. Designation “mod/mod” refers to transgenic lines homozygous for the mod(mdg4)u1 or mod(mdg4)T6 mutation. An asterisk indicates variegated eye pigmentation. Other designations are as in Figure S1 and S2. (TIF) [file pgen.1003606.s003.tif]

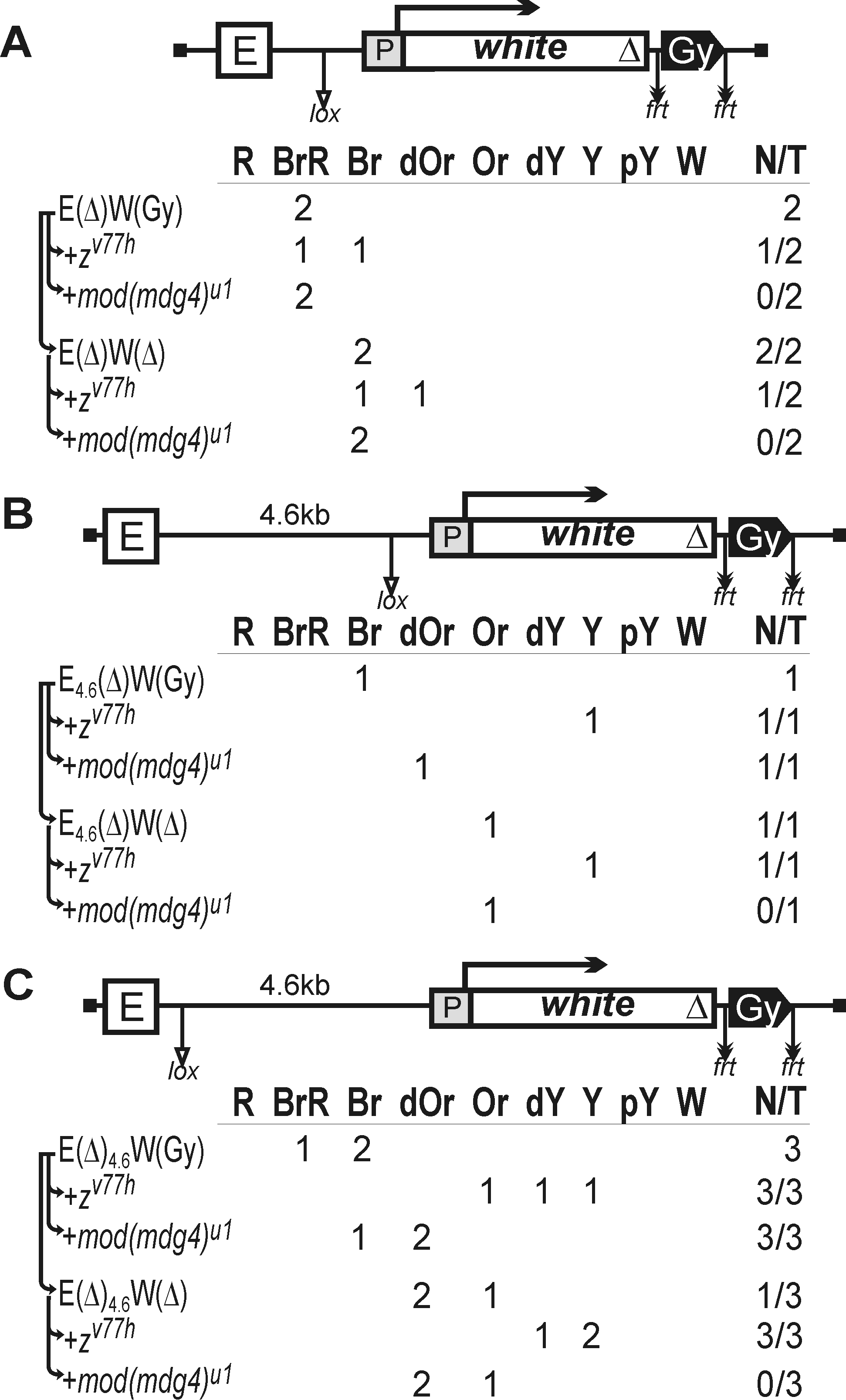

Supplement: Figure S4 — Role of the gypsy insulator located on the 3′ side of the white gene and Mod(mdg4)-67.2 in stimulation of white expression. The derivatives carrying one copy of the gypsy insulator from the 3′ side of the white gene were tested. The original and derivatives transgenic lines are described in Figure 6. In (A) the enhancer is located near the white gene. In (B) and (C) the enhancer is located at the 4.6 kb distance from the promoter. Designations are as in Figure S1 and S3. (TIF) [file pgen.1003606.s004.tif]

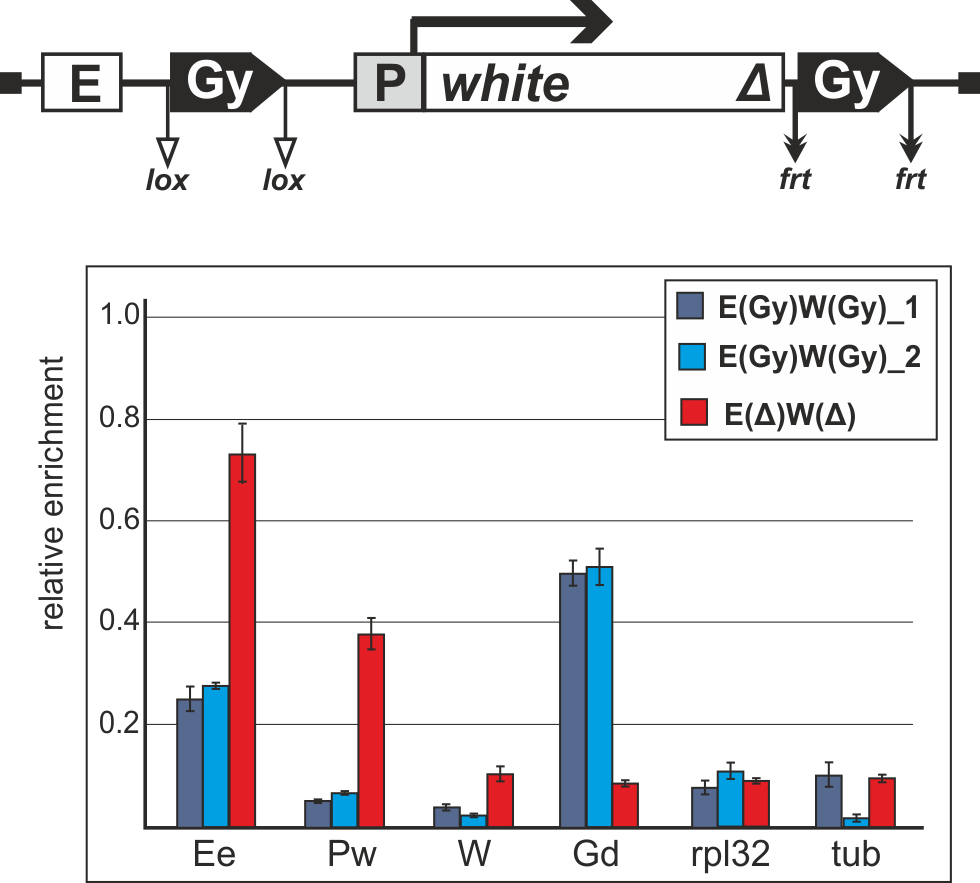

Supplement: Figure S5 — The results of ChIP of specified chromatin regions with antibodies to Zeste in two transgenic lines carrying constructs with two copies of the gypsy insulators. Designations: Ee (the eye enhancer), Pw (promoter), W (coding region of the white gene) and Gd (distal gypsy insulator). The rpl32 and tubulin (tub) coding regions were used as controls devoid of Zeste binding sites. Other designations are as in Figure S1. (TIF) [file pgen.1003606.s005.tif]

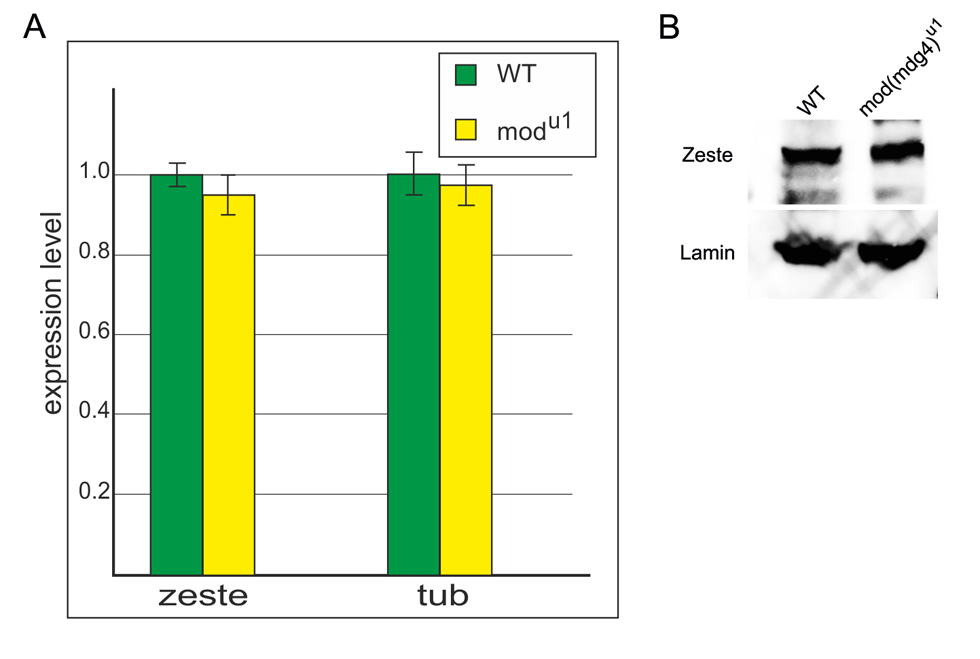

Supplement: Figure S6 — Testing for the direct influence of the mod(mdg4)u1 mutation on the expression of Zeste. (A) Relative levels of the zeste gene expression in wild-type (WT) and mod(mdg4)u1 backgrounds. The transcripts were isolated from 2-day pupae and quantified by RT-PCR, with tubulin (tub) expression being used as a control. The transcript levels were normalized relative to that of rpl32. Error bars standard deviations of triplicate measurements. (B) Levels of the Zeste protein in wild-type (WT) and mod(mdg4)u1 pupae tested by Western blot analysis. Lamin was used as a loading control. (TIF) [file pgen.1003606.s006.tif]

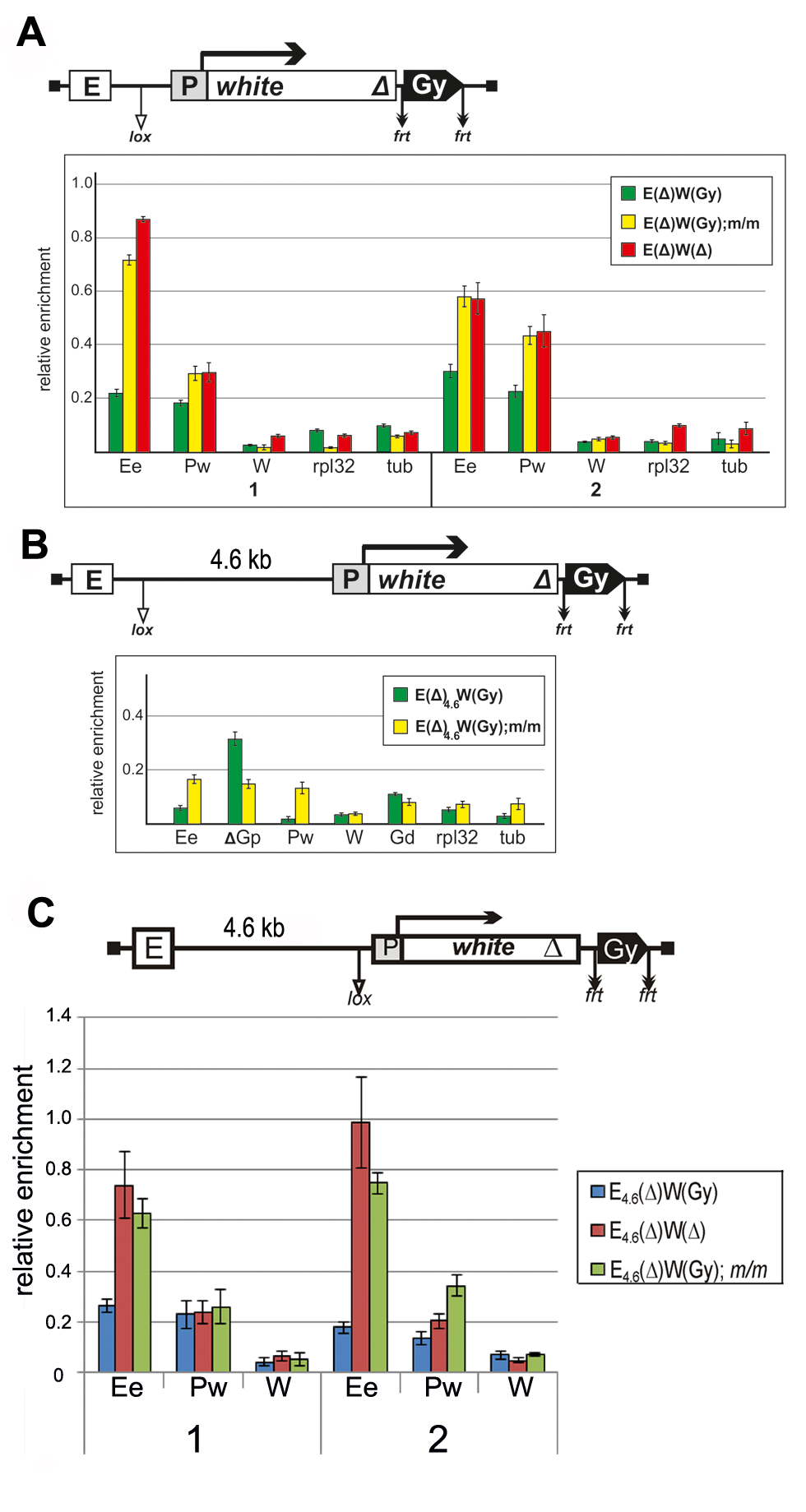

Supplement: Figure S7 — The effect of Mod(mdg4)-67.2 on the level of Zeste in transgenic lines. (A) The results of ChIP (percentages of input DNA normalized relative to the endogenous positive binding site for Zeste from the Ubx promoter region) of specified chromatin regions with antibodies to Zeste in derivatives of two transgenic lines carrying the eye enhancer in close proximity to the white promoter in the wild-type and mod(mdg4)u1 (m/m) mutant backgrounds. Phenotypes of these derivative lines are described in Figure S4A. (B) The results of ChIP with antibodies to Zeste in derivatives of the transgenic line described in Figure 8 in the wild-type and the mod(mdg4)u1 backgrounds. Phenotypes corresponding for these lines are described in Figure S4B. (C) The results of ChIP with antibodies to Zeste in derivatives of two transgenic line carrying the eye enhancer at 4.6 kb from the white promoter in the wild-type and the mod(mdg4)u1 backgrounds. Phenotypes corresponding to these lines are described in Figure S4C. Other designations are as in Figures S4 and S5. (TIF) [file pgen.1003606.s007.tif]
