# Supplementary material for: Effective Blocking of the White Enhancer Requires Cooperation between Two Main Mechanisms Suggested for the Insulator Function
Source: PLoS Genet. 2013 Jul 4;9(7):e1003606. doi: 10.1371/journal.pgen.1003606 (PMC3701704; doi:10.1371/journal.pgen.1003606)
Supplement: Table S1 — Oligos used in the study. Table summarizes information about oligos used in ChIP, 3C and RT-PCR experiments. (DOC) [file pgen.1003606.s008.doc]

| **Primer** | **Sequence** | **Application** |
| --- | --- | --- |
| Ee_fw | 5'-gcgacaggcgagtgacaataa-3' | ChIP |
| Ee_rev | 5'-ttggttgagtgaagctcgaatc-3' | ChIP |
| Pw_fw | 5'-gcactggatatcattgaacttatctg-3' | ChIP |
| Pw_ rev | 5'-tggacagagaaggaggcaaaca-3', | ChIP |
| W_fw | 5'-gcaaatgtcagcacacgatcat-3' | ChIP |
| W_ rev | 5'-gtgggctcatcgcagatca-3', | ChIP |
| rpl32_ fw | 5'-gttcgatccgtaaccgatgt-3' | ChIP, RT-PCR |
| rpl32_ rev | 5'-ccagtcggatcgatatgctaa-3', | ChIP, RT-PCR |
| tub_fw | 5'-gctttcccaagaagctcataca-3' | ChIP, RT-PCR |
| tub_ rev | 5'-ggttcagtgcggtattatccag-3' | ChIP, RT-PCR |
| Gp_fw | 5'-tcgactctactagaaggcctaa-3' | ChIP, 3C |
| Gp_rev | 5'-ggcatggtgccaatgaatc-3' | ChIP, 3C |
| Gd_fw | 5'-gggatccttgaagttcctattc-3' | ChIP |
| Gd_rev | 5'-aattcgaggtcgacgatagc-3' | ChIP |
| Ubx_fw | 5'-﻿﻿tccttcctcgcgaatgaatgaacg-3'﻿﻿ | ChIP |
| Ubx_ rev | 5'-tcgaacgaatgtggcaccaaatc-3'﻿ | ChIP |
| Zeste_fw | 5'-cgttaaggtggaacccgagtac-3'﻿ | RT-PCR |
| Zeste_rev | 5'-cacatcgttggcatccatctc-3'﻿ | RT-PCR |
| 1_fw | 5'-gaaagcggtgaggtctctaa-3'﻿ | 3C |
| 2_fw | 5'-cccacaaaggaagtctcttac-3'﻿ | 3C |
| 3_fw | 5'-gctgagtgagacatcgaattg-3'﻿ | 3C |
| 3_probe | 5'-(FAM)atcgaattgggcgagctcgaattagg(BHQ-1)-3'﻿ | 3C |
| 4_fw | 5'-gcttgatagcttgatagcttga-3'﻿ | 3C |
| 5_fw | 5'-ttcgacgttcagacgtagtg-3'﻿ | 3C |
| 6_fw | 5'-cgtgatggtaagtggaagttaaa-3'﻿ | 3C |
| 7_fw | 5'-gccaccagccattgaataata-3'﻿ | 3C |
| 8_fw | 5'-ctgcactggatatcattgaactta-3'﻿ | 3C |
| 9_fw | 5'-aaactcctctcgcttcttattt-3'﻿ | 3C |
| 9_probe | 5'-(FAM)atcctcttggcccattgccgggattt(BHQ-1)-3'﻿ | 3C |
| 10_fw | 5'-gctgtgctcgctatataagac-3'﻿ | 3C |
| 11_fw | 5'-ggagaatgggtacacctaca-3'﻿ | 3C |
| 12_fw | 5'-gacatatatccgaaataactgcttg-3'﻿ | 3C |
| 12_probe | 5'-(FAM)agggggcaataaacagtaaacacgatg(BHQ-1)-3'﻿ | 3C |
| 13_fw | 5'-agcttatcgataccgtcgag-3'﻿ | 3C |
| 14_fw | 5'-gataaagcggagacacgaaag-3'﻿ | 3C |
| 15_fw | 5'-tgctggatgttctttgagttt-3'﻿ | 3C |
